# Poor sleep quality, dementia status and their association with all-cause mortality among older US adults

**DOI:** 10.18632/aging.206102

**Published:** 2024-09-04

**Authors:** May A. Beydoun, Rio Tate, Michael F. Georgescu, Alyssa A. Gamaldo, Christian A. Maino Vieytes, Hind A. Beydoun, Nicole Noren Hooten, Michele K. Evans, Alan B. Zonderman

**Affiliations:** 1 Laboratory of Epidemiology and Population Sciences, National Institute on Aging, Intramural Research Program, NIA/NIH/IRP, Baltimore, MD 21224, USA; 2 Department of Psychology, Clemson University, Clemson, SC 29634, USA; 3 Department of Veterans Affairs, VA National Center on Homelessness Among Veterans, Washington, DC 20420, USA; 4 Department of Management, Policy, and Community Health, School of Public Health, University of Texas Health Science Center at Houston, Houston, TX 77030, USA

**Keywords:** sleep quality, dementia, mortality, aging, cohort studies

## Abstract

Background: Evidence points to associations between sleep quality, dementia, and mortality. We examined whether poor sleep quality mediated or moderated the association between dementia and mortality risk among older US adults and vice versa, and whether these associations differed by sex and by race.

Methods: The study investigated bi-directional associations between sleep quality, dementia and mortality in older US adults using data from the Health and Retirement Study (*N* = 6,991, mean age = 78.1y, follow-up: 2006–2020, number of deaths = 4,938). It tested interactions and mediating effects, using Cox proportional hazards models and four-way decomposition models.

Results: Poor sleep quality was associated with increased mortality risk, particularly among male and White older adults. However, the association was reversed in the fully adjusted model, with a 7% decrease in risk per tertile. Probable dementia was associated with a two-fold increase in mortality risk, with a stronger association found among White adults. The association was markedly attenuated in the fully adjusted models. Sleep quality-stratified models showed a stronger positive association between dementia and mortality among individuals with better sleep quality. Both mediation and interaction were involved in explaining the total effects under study, though statistically significant total effects were mainly composed of controlled direct effects.

Conclusions: Poor sleep quality is directly related to mortality risk before lifestyle and health-related factors are adjusted. Dementia is linked to mortality risk, especially in individuals with better sleep quality, males, and White older adults. Future research should explore the underlying mechanisms.

## INTRODUCTION

Inadequate sleep duration and poor sleep quality are becoming significant public health issues linked to cardiometabolic risk factors like obesity, particularly with an aging population [1]. Approximately 20% of adults are impacted by health issues associated with substandard sleep quality or insufficient sleep durations [2, 3]. Simultaneously, maintaining a healthy cognitive function is necessary for carrying out essential everyday tasks, such as remembering to take prescribed medication or prepare meals [4, 5]. Attention, working, short-term or long-term memory, motor coordination and reasoning all have an impact on cognition [4, 5]. As individuals get older, the occurrence of brain disorders that may affect cognitive abilities, such as stroke and dementia, becomes more common [4, 5]. This can engender obstacles for people when it comes to taking on and completing daily chores [4, 5]. Dementia is characterized by a decline in overall cognitive functioning across several areas, as well as the inability to perform routine activities [4–6]. The prevalence of dementia among individuals aged 60 and above is estimated to be 4.7%, with an annual increase of 4.6–7.7 million new cases globally (equivalent to 3.5–10.5 cases per 1,000 individuals) [5, 7–9].

More importantly, research has demonstrated that the occurrence of dementia is indicative of a greater risk of future all-cause mortality (e.g., [10]). Furthermore, there is increasing evidence suggesting that both short and lengthy sleep durations, as well as other disturbances, are associated with higher risks of mortality from all causes (e.g., [3, 11]). In addition, there have been limited attempts in prospective cohort studies to assess the chronological connection between sleep and neurodegenerative illnesses, such as dementia and age-related cognitive decline (e.g., [12–15]). The studies usually found that insufficient sleep length, low sleep quality, and sleep disorders were associated with negative outcomes indicating impaired cognitive aging, including various dementing illnesses. In addition, there is further evidence suggesting that preclinical dementia can impact sleep patterns and disorders (e.g., [16]).

Considering the interconnection between sleep, dementia, and the rate of mortality, it is important to investigate the pathways and potential interactions among them as significant research inquiries. However, a systematic study has been lacking to date that has thoroughly investigated the mediating and moderating association between sleep, dementia, and the risk of death in older persons, especially in nationally representative samples. It is crucial as well to analyze those relationships within different sex and ethnic categories. Existing research reports associations between sleep and dementia, dementia and mortality, and sleep and mortality among older persons. However, there are no studies that specifically investigate the bi-directional pathways that link sleep quality, dementia, and mortality risk.

This study aims to investigate the relationship between poor sleep quality and dementia status with mortality risk. We examine this relationship independently of potential confounding factors, while also considering the influence of sex and race. The study is conducted using a sub-sample of the Health and Retirement Study (HRS) with complete algorithmically defined dementia status and probability outcomes. The participants in this sub-sample have a mean age of approximately 78 years. Furthermore, we conduct a simultaneous examination to assess the potential interaction between poor sleep quality and dementia outcomes in determining the risk of mortality. We also investigate the bidirectional mediation effects of “poor sleep quality” and “dementia status” on mortality, with dementia status mediating the relationship between poor sleep quality and mortality, and poor sleep quality mediating the relationship between dementia status and mortality. To analyze these relationships, we employ four-way decomposition models.

## METHODS

### Database

The HRS is an ongoing study that follows a group of adults in the United States who are 50 years of age or older [17, 18]. The survey has been conducted every two years since 1992 [17, 18]. The HRS is being funded by the National Institute on Aging (grant number U01AG009740) and the Social Security Administration [17, 18]. Previous publications have provided comprehensive explanations of HRS approaches [17–20]. In 2006, HRS introduced the Enhanced Face-to-Face Interview (EFTF), which includes anthropometric measurements, physical performance tests, blood and saliva samples, and self-administered questionnaires covering among other psychosocial domains of interest [17, 18]. Out of all the primary sample units (PSUs), almost 50% of the houses that had at least one resident responder were selected for the EFTF interview [17, 18].

An initial EFTF interview was carried out with a randomly selected half-sample of participants from the 2006 HRS wave [17, 18]. In 2008, the second half sample was subsequently interviewed [17, 18]. Similarly, in 2010, households within the same cohort were randomly assigned to one of these two groups, and the collection of EFTF data started either in 2010 or 2012 [17, 18]. In order to ensure that both members of a family received the identical request, the sample was selected at the household level [17, 18]. The newly married partners of those who were selected for the EFTF interview were also requested to participate in the same process [17, 18]. Consequently, in households when the members are coupled, both individuals in the pair were selected [17, 18]. Certain participants in the EFTF study were exempt from completing the physical measurements or biomarker assessments [17, 18]. The individuals in this group were required to be interviewed through a proxy, resided in nursing homes, or refused a face-to-face interview but consented to being interviewed over the phone [17, 18].

### Standard protocol approvals, registrations, and patient consents

The Institutional Review Board (IRB) at the University of Michigan, Ann Arbor (NIA U01 AG009740; URL: Institutional Review Board Information (umich.edu)) granted approval after the procedures were carried out in compliance with the institution or the regional committee on human experimentation’s ethical standards [17, 18]. The ethics board found that there was no need for or waiver of participant consent [17, 18]. The National Institutes of Health’s Intramural Research Program granted approval for the current retrospective analysis of the parent IRB-approved study, which was deemed to be research not involving human subjects [17, 18].

### Study sample

Starting from the 43,561 HRS participants (1992–2018) that were initially included in the longitudinal RAND file, 18,469 were still alive in 2006 and included in the study; of them, 17,809 were older than 50 years (Supplementary Figure 1). Of this sub-sample, 7,115 had information on their dementia status in 2006. After accounting for missing data on the sleep quality scale, the final selected sample consisted of up to 6,991 persons who were over 50 years old, with simultaneous availability of dementia and sleep quality data. These participants were followed up until the end of 2020 to determine their mortality status from all causes. The final sample’s mean age in 2006 was 78 years old, given the exclusion of participants without dementia status data, resulting in an age range of 60–104 years old.

### Mortality from all causes

**Figure 1:**
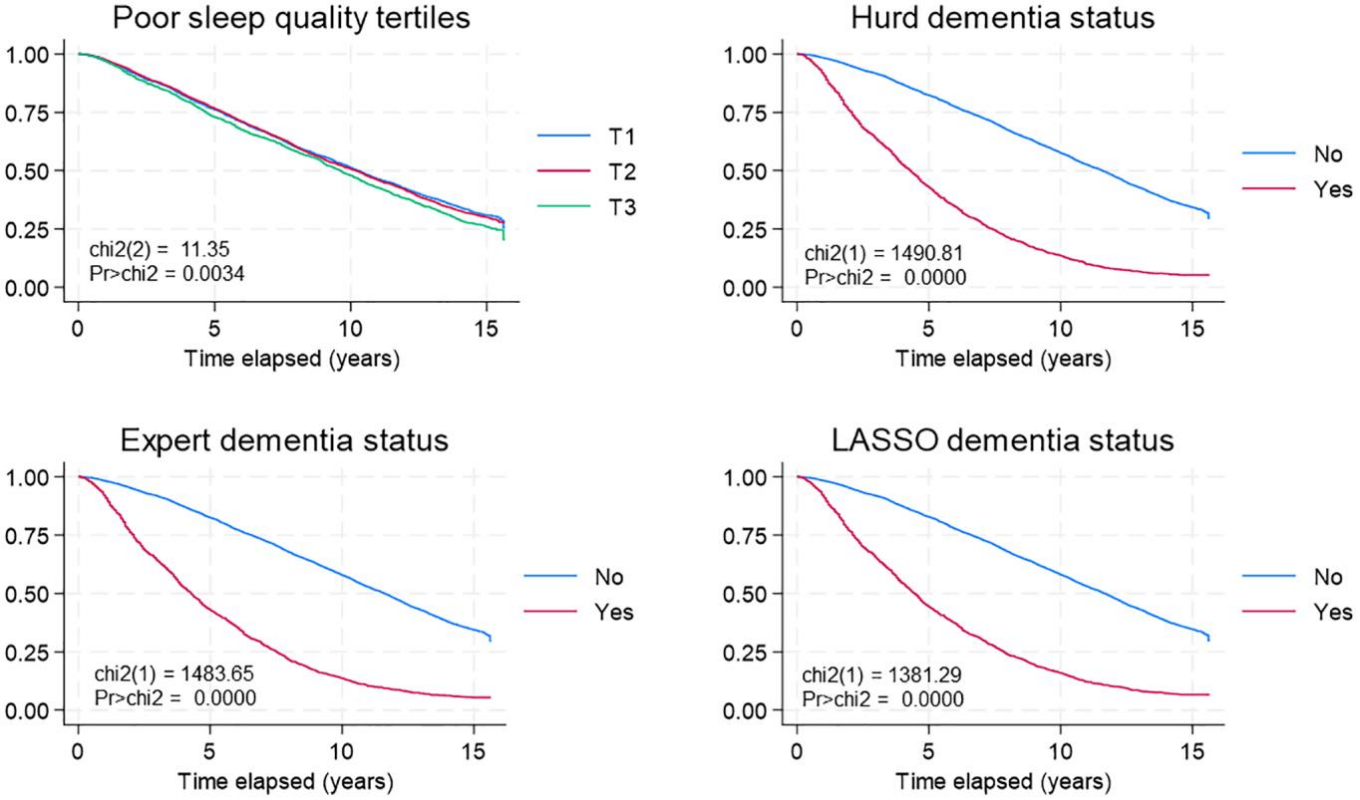
**Sleep quality, dementia status and all-cause mortality: K-M survival curves.** Abbreviations: chi2: Chi-square statistic; HRS: Health and Retirement Study; K-M: Kaplan Meier; LASSO: Least Absolute Shrinkage and Selection Operator; Pr: Probability.

In this study, the time to all-cause death between 2006 and 2020 was the outcome variable. The most recent measurements of exposures and mediators (2006 for both) were used to follow-up on all-cause mortality. By linking data from the population register and interviewing informants or other knowledgeable individuals, the HRS was able to identify and ascertain deaths [21]. Tracker file variables were utilized which were updated to 2020 (v2). Of the 6,991 individuals chosen for our final analytic sample, 4,938 deaths occurred during the follow-up period of 2006–2020 from any cause, with a known approximate date of death (month and year). The period between the approximate age at death and the age at examination in 2006, which was calculated at the end of the wave, was used to determine the time to death [18]. Years were used to quantify the passage of follow-up time. This period of follow-up for those who survived to the end of 2020 was roughly 15 years. Overall, the eligible sample’s mortality rate was estimated at 74 per 1000 P-Y (Figure 1).

### Dementia occurrence measures

Similar to a previous study [18], the dataset file named “hrsdementia_2021_1109.sas7bdat” is accessible to the public and includes probabilities and classifications related to dementia. These data presented pertain to individuals aged 60 and above who participated in the 2006 survey. The respondents were categorized as such if their self-reported race/ethnicity was “non-Hispanic white”, “non-Hispanic black”, or “Hispanic”. Three recently created algorithms were utilized: a modified iteration of an algorithm initially devised by Hurd and colleagues (Modified Hurd Algorithm), a novel logistic model informed by experts (Expert Algorithm), and a Least Absolute Shrinkage and Selection Operator (LASSO) algorithm. These algorithms were trained and tested using data from the Health and Retirement Study (HRS) as well as data from all four waves of the Aging, Demographics, and Memory Study (ADAMS; http://hrsonline.isr.umich.edu/index.php?p=shoavail&iyear=XB). The models demonstrated a sensitivity range of 77–83%, specificity range of 92–94%, and an overall out-of-sample performance accuracy range of 90–92%.

We will briefly include some cursory information regarding each probability algorithm, but we refer the reader to a technical appendix made available in an earlier study [18]. The Modified Hurd Algorithm comprised training an ordered probit model on the ADAMS subset of participants with cognitive testing data, which were obtained through the Telephone Interview for Cognitive Status (TICS) or the Informant Questionnaire on Cognitive Decline in the Elderly (IQCODE). The probit model included a vector of prespecified participant characteristics and variables. It was then used to predict the probability of cognitive status outcomes (an ordinal factor—*dementia*, *cognitive impairment but not dementia*, and *aging normally*) for each subject. For a given subject, the outcome with the highest probability was the dementia classification assigned to that individual. The Expert Algorithm used a logistic regression model trained on ADAMS subjects, in a similar fashion as the Modified Hurd Algorithm, to classify subjects into *dementia* and *no dementia* categories. Classification of subjects into those categories used race-specific probability thresholds that maximized the sensitivity and specificity and were obtained via 10-fold cross-validation. Finally, the LASSO algorithm was similar to the Expert Algorithm with the exception that the trained logistic regression model for dementia classification included LASSO penalization. Citations for these methods are available in the Supplementary Materials (Supplementary Method 1).

### Poor sleep quality

The assessment of poor sleep quality was conducted using a set of 5 items, with 4 items scored on a Likert scale ranging from 1 to 3, and one item measured as a binary outcome. The items KC083 (insomnia), KC084 (nocturnal awakenings), KC085 (early morning awakenings), and KC086 (feeling rested in the morning) can be found in section C of the physical health questionnaire for the 2006 wave of data (public use data, wave K). A supplementary binary measure, coded as KC232U2, indicates the use of medication for sleep. More information can be found at this URL: https://hrs.isr.umich.edu/sites/default/files/meta/2006/core/codebook/h06c_ri.htm. The items were subjected to reverse coding in order to reliably represent lower sleep quality with higher scores (1 = good quality, 2 = medium quality, 3 = poor quality), except for medication use (0 = no, 1 = yes). Next, the five components were added together and adjusted to a new scale that ranges from 0 to 9. Additional information can be found in Supplementary Method 2.

### Covariates

#### Socio-demographic factors

Our study took into account various socio-demographic factors, such as gender (male, female), age as of 2006, race (White vs. Black/Hispanic/Others), marital status (never married, married/partnered, separated/divorced, widowed), education (no degree, general educational development, high school graduate, some college, college degree or higher), employment status (working, not working), and total wealth (in US dollars) (<25,000, 25,000–124,999, 125,000–299,000, 300,000+), as was done in previous studies [17, 18, 22]. Elsewhere, a review has examined the connections between socioeconomic characteristics and sleep, dementia risk, and mortality [23]. The evidence suggests that lower socioeconomic status has unfavorable consequences on these outcomes.

#### Lifestyle factors

The lifestyle metrics that were taken into account were the individual’s smoking status (categorized as never smoker, past smoker, or current smoker) and the frequency of moderate/vigorous activity (categorized as never, 1–4 times per month, or more than once per week) [17, 18, 22]. Elsewhere, a review examines the correlation between these specific lifestyle characteristics and sleep, dementia risk, and mortality [24–27]. These studies also suggest that making poor lifestyle choices, such as engaging in less physical activity and smoking more frequently, is connected with negative consequences.

#### Health-related factors

Participants were asked to rate their health as either “excellent/very good/good” or “fair/poor” [17, 18, 22]. In addition, the HRS collected self-reported data on weight and height, as well as information on the presence of cardiometabolic risk factors and chronic illnesses, as diagnosed by a physician [17, 18, 22]. The HRS inquired about depressed symptoms using an 8-item Centers for Epidemiologic Studies-Depression scale total score [17, 18, 22]. Prior studies found that all four health-related factors were linked to an increased risk of mortality, lower quality of sleep, and a higher incidence of dementia [26, 28–31]. With the exception of unchangeable elements like sex and race, any other socio-demographic, lifestyle, and health traits that were assessed in 2006 were regarded as confounding variables [17, 18, 22]. Moreover, to increase the sample size, these components underwent multiple imputation [17, 18, 22].

### Statistical methods

Through the use of Stata 18.0 (StataCorp, College Station, TX, USA), [32] our analyses took sampling design complexity into consideration through the inclusion of sampling weights, primary sampling units, and strata for most analyses. Aside from outcomes, covariates such as major exposures, mediators, and moderators were imputed through the use of chained equations when missing data were detected starting with the sample aged over 50 y in 2006 (*n* = 17,938) [17, 18, 22, 33]. Missing data proportion in this sample was <2%, ranging from *n* = 0 in 6 covariates to *n* = 185 in one covariate out of a total of 13 imputed covariates. The main commands used to this end were mi impute followed by mi passive and mi estimate, as well as mi svyset and mi stset [18, 34]. Detailed code is provided on https://github.com/baydounm/HRS_SLEEP_DEMENTIA_MORTALITY_SUPPLEMENT. We further utilized survey (svy) commands to estimate population proportions, means, in addition to regression coefficients, while standard errors (SE) were adjusted through Taylor series linearization [17]. Analyses were performed in aggregate, and stratification was further performed by sex and race [18]. The svy:reg and svy:mlogit commands were used for comparisons of means and proportions of key measures by sex and race groups, using sex and racial contrasts as the sole predictors in these models [18].

We established the time-to-event (measured in years) starting from the age of entrance being greater than 50 years (referred to as delayed entry) and continuing until the exit age when either the event of interest (all-cause deaths) or censoring (loss to follow-up or termination of follow-up) took place [17, 18]. Loss to follow-up refers to the situation where a respondent, who was alive and participated in the previous wave, does not take part in a subsequent wave [17, 18]. The time variable utilized in the analysis was the number of years between the estimated age by the end of 2006 and the estimated event or censoring age [17, 18]. Only participants who were living in 2006 were included in the final analysis [17, 18]. Survival curves using the Kaplan-Meier method were implemented for different levels of poor sleep quality and different groups based on dementia status [17, 18]. The equality of survivor functions was assessed between groups using a log-rank test (specifically, the *sts* test with the logrank option) [17, 18].

In order to evaluate our main assumptions, we conducted Cox proportional hazards models for mortality risk [17, 18]. These models were stratified by race and sex, and were performed on imputed data. The models included socio-demographic, SES, lifestyle, and health-related variables. Two models were used: a reduced model (Model 1) and a fully adjusted model (Model 2) [17, 18]. The proportionality of the risks assumption was checked for each model. Hazard ratios, together with associated standard errors, were computed using multiple imputed data. Model 1 consisted of age in 2006, sex, and race as independent variables. Model 2 included additional covariates to this simplified model, specifically education, total wealth, marital status, smoking status, physical exercise, self-rated health, body mass index categories, cardiometabolic risk groups, and continuous total CES-D score. The primary variables of interest in these models were the continuous measure of “poor sleep quality” and the “dementia probability” converted using the Log_e_odds, with odds being the Pr/(1-Pr) transformation, as applied to the Hurd, expert, and LASSO algorithms (Analysis A) [18]. The study additionally examined the variation in the impact of “poor sleep quality” and “Loge (dementia odds)” on the risk of death based on gender and race [18]. This was done by introducing interaction terms between these factors and gender or race, respectively [18]. An analogous analytical method was utilized to examine the relationship between binary dementia status factors and sleep quality tertiles, which were the primary variables of interest in Analysis B [18]. An increase of one unit in the Log_e_(odds) of the dementia outcomes equates to a shift in the probability of dementia from 0% to 73% [18].

To examine the relationship between the likelihood of developing dementia and mortality risk based on sleep quality tertiles, further models were implemented. These models, referred to as the reduced model (Model 1) and the fully adjusted model (Model 2), followed a similar procedure as previously described. More precisely, each dementia exposure metric was individually included in Cox proportional hazards models to analyze the relationship with overall mortality. The analysis was stratified by sleep quality tertiles. The study additionally examined differentials across sleep quality across tertiles by including 2-way interaction terms with each alternative dementia exposure in the unstratified model. Each Ln(odds) of dementia utilizing the Hurd, expert, and LASSO algorithms, was included as a potential mediator/moderator in the relationship between poor sleep quality and the risk of mortality from 2006 to 2020. This aspect of the analysis was likewise conducted on multiple imputed data, although the complexity of the sample design was not taken into consideration. This portion of the analysis aimed to analyze the impact of “poor sleep quality” on mortality risk, taking into account the potential interaction with a mediator. The analysis decomposed the overall effect into four distinct components: (i) the controlled direct effect (CDE), which represents the effect without mediation or interaction; (ii) the interaction referent (INTREF), which represents the effect of interaction alone without mediation; (iii) the mediated interaction (INTMED), which represents the effect of both mediation and interaction; and (iv) the pure indirect effect (PIE), which represents the effect of mediation without interaction, as was done elsewhere [6, 17, 18, 22, 34, 35]. This four-way decomposition consolidates approaches for comprehending the effects and the comparative constitution of those effects within the framework of the four previously mentioned components (Supplementary Method 3). Stata has just implemented this method, which enables the estimation of parametric or semi-parametric regression models with four-way decomposition. The Med4way command [36] (https://github.com/anddis/med4way) was utilized to examine the mediation and interaction of the overall impact of the “poor sleep quality” exposure on the all-cause mortality outcome. This analysis considered three transformed “dementia probability” measures as potential alternative mediator/moderator variables. Cox proportional hazards models were used for the outcome, and ordinary least squares regression models were used for each mediator/moderator. The complete sample underwent a four-way decomposition, utilizing age in 2006, sex, and race as exogenous factors. Additionally, the decomposition was stratified by both sex and race. The complete model was also regarded as a sensitivity analysis. Additionally, a 4-way decomposition was performed to analyze the relationship between Log_e_ (dementia odds) and mortality risk (“dementia-mortality” analysis). This analysis involved examining the potential mediators of poor sleep quality, using the same analytical method as the “sleep-mortality” analysis. Exposures and mediators/moderators were normalized using a Z-score transformation in all four-way decomposition models. The significance level for all analyses was established at 0.05, which means that the probability of making a Type I error was set at 0.05. The significance level for Type I error in the analysis of 2-way interaction variables involving sex and race was established at 0.10 [37].

Additionally, several secondary and sensitivity studies were conducted. Initially, a different metric for sleep quality was used, which excluded the item related to the use of sleep medicines. The results of these studies are included in the supplemental materials available online. Additionally, we conducted a sub-analysis that examined the relationship between sleep quality measures and mortality, taking into account alcohol use as one of the factors considered. Furthermore, each item of the sleep quality score was recoded as follows: 1 = good sleep quality, 2 = medium sleep quality, and 3 = poor sleep quality. These recoded scores were then assessed against the outcomes of all-cause mortality and dementia status using Cox proportional hazards and logistic regression models, respectively. The analysis was performed on multiple-imputed data. Only bivariate models are shown for the final selected sample. Ultimately, the fully adjusted model underwent backward elimination to investigate the impact of various confounders on the link between poor sleep quality and mortality. This process aimed to find the most influential confounder(s) in this relationship. The primary Stata output and the results of the sensitivity analysis may be found at: (refer to https://github.com/baydounm/HRS_SLEEP_DEMENTIA_MORTALITY_SUPPLEMENT), and the general analytical approach was adopted in other recent studies [6, 17, 18, 22, 34, 35].

### Data availability

While the data are owned by the University of Michigan Ann Arbor and Health and Retirement Study (HRS) is public use data, this work is owned and funded by the National Institute on Aging at the NIH. Scripts used in this analysis will be made available on a github repository: https://github.com/baydounm/HRS_SLEEP_DEMENTIA_MORTALITY_SUPPLEMENT. For additional information please contact the corresponding author by e-mail contact at baydounm@mail.nih.gov.

## RESULTS

According to Table 1, male and Black/Hispanic/Others selected participants were on average younger than their female and White counterparts. Generally, females had lower socio-economic status, in terms of lower educational attainment, belonging to lower income categories, and reporting non-working status. Moreover, females were more likely to be widowed compared to their male counterparts. On the other hand, females were more likely to be never smokers, to be in the normal BMI range, and less likely to report ≥3 cardio-metabolic factors, even though they were less physically active. Females had a higher CES-D score, on average compared to males, as well as a poorer sleep quality score, and higher algorithmically defined dementia probability and proportion. Notable racial differences were observed as well. In fact, Black/Hispanic/Others participants had lower socio-economic status, based on education, income and work status compared to their White counterparts. They were also more likely to be divorced/separated or widowed, with higher proportion of current smokers, and lower percentage reporting physical exercise > 1 time/week. Black/Hispanic/Others individuals were more likely to be obese (30% vs. 21%) and had on average a higher CES-D total score, although they reported better sleep quality compared their White counterparts. Dementia probability was significantly greater among Black/Hispanic/Others compared to White participants. Mortality rate was significantly greater among males compared to females though differences by race were not detected.

**Table 1:** Study sample characteristics: overall, by sex and by race/ethnicity groups: HRS 2006–2020^a^.

	**Overall**	**Male**	**Female**	**White**	**Black/Hispanic/ Others**	**P^b^_sex_**	**P^b^_race_**
	**Mean ± SEM, %**	**Mean ± SEM, %**	**Mean ± SEM, %**	**Mean ± SEM, %**	**Mean ± SEM, %**		
Unweighted *N*	*N* = 6,758	*N* = 2,869	*N* = 3,889	*N* = 5,471	*N* = 1,287		
***Sex, % male***	41.2	100	0	41.7	38.1	n/a	0.012
***Age (years), 2006***	78.1 ± 0.1	77.6 ± 0.1	78.5 ± 0.1	78.3 ± 0.1	77.3 ± 0.2	<0.001	<0.001
***Race, % Black/Hispanic/ Others***	13.5	12.5	14.2	0	100	0.012	n/a
***Education:***							
No degree	25	25.7	24.5	20.1	56.4	<0.001	<0.001
General Educational Development	3.9	4.8	3.2	3.8	4.4	<0.001	<0.001
High School graduate	33.7	27	38.4	35.6	21.6	(Ref)	(Ref)
Some college	19	17.4	20.1	20.2	11.3	0.025	0.44
College degree or higher	18.5	25.1	13.8	20.4	6.3	<0.001	<0.001
***Marital status:***							
Never married	2.5	2.5	2.5	2.3	4.1	<0.001	<0.001
Married/Partnered	53.8	74.3	39.4	55.3	44.3	(Ref)	(Ref)
Separated/Divorced	7.7	6	8.9	7	11.9	<0.001	<0.001
Widowed	36	17.2	49.2	35.4	39.6	<0.001	<0.001
***Work status:***							
Not Working	87.2	82.4	90.5	86.8	89.4	(Ref)	(Ref)
Working	12.8	17.6	9.5	13.2	10.6	<0.001	0.097
***Total wealth ($):***							
<25,000	44.2	31.1	53.2	39.7	72.4	<0.001	<0.001
25,000–124,999	51	61.6	43.6	54.7	27.2	(Ref)	(Ref)
125,000–299,999	4	6	2.8	4.7	0.3	<0.001	<0.001
≥300,000	0.8	1.3	0.6	0.9	0.1	0.001	<0.001
***Smoking status:***							
Never smoker	44.2	28.8	55	43.8	47.2	<0.001	0.015
Past smoker	48.6	64.1	37.8	49.3	44.6	(Ref)	(Ref)
Current smoker	7.1	7.1	7.2	7	8.2	<0.001	0.048
***Frequency of PE:***							
Never	27.2	20	32.3	26.1	34.6	<0.001	<0.001
1–4 times per month	18.8	19.2	18.5	18.3	22	0.002	<0.001
>1 times per week	54	60.8	49.2	55.7	43.4	(Ref)	(Ref)
***Self-rated health:***							
Excellent/very good/good	67.4	68.1	66.8	70.5	47.4	(Ref)	(Ref)
Fair/poor	32.6	31.9	33.2	29.5	52.6	0.37	<0.001
***Body mass index (kg/m^2^):***							
*** <25***	39.5	32.5	44.5	41	30.1	(Ref)	(Ref)
*** 25–<30***	38.1	45.7	32.8	37.9	39.6	<0.001	<0.001
*** ≥30***	22.3	21.8	22.7	21.1	30.2	<0.001	<0.001
***Number of cardiometabolic factors and conditions* ^c^: **							
*** 0***	24.9	23.5	25.9	25.5	21	0.32	0.004
*** 1–2***	63.4	62.6	64	63	65.7	(Ref)	(Ref)
*** ≥3***	11.7	14	10.1	11.5	13.3	<0.001	0.28
***CES-D total score***	1.48 ± 0.03	1.18 ± 0.04	1.68 ± 0.04	1.40 ± 0.03	1.97 ± 0.07	<0.001	<0.001
***Mortality rate, 2006–2020***							
# deaths per 1000 P-Y with 95% CI	73.8	81.1	68.9	73.9	73.3	n/a	n/a
	(71.8−75.9)	(77.8−84.6)	(66.4−71.6)	(71.7−76.3)	(68.8−78.3)		
**Poor sleep quality score**	2.78 ± 0.03	2.46 ± 0.05	3.01 ± 0.03	2.80 ± 0.03	2.66 ± 0.06	<0.001	<0.001
**Dementia status, % yes**							
Hurd	14.2	12.8	15.3	13.5	19.3	0.013	<0.001
Expert	14.9	12.6	16.5	13.8	21.7	0.001	<0.001
LASSO	16	13.8	17.5	14.8	23.9	0.001	<0.001
**Dementia probability**							
Hurd	0.1	0.09	0.11	0.09	0.17	0.001	<0.001
Expert	0.13	0.11	0.14	0.12	0.2	<0.001	<0.001
LASSO	0.13	0.11	0.15	0.12	0.19	<0.001	<0.001

Abbreviations: HRS: Health and Retirement Study; PE: physical exercise; SEM: Standard Error of the Mean. ^a^Values are means ± SE or % ± SE, overall and across sex or race/ethnicity groups for main baseline and fixed sample characteristics (see Covariates section for detail), taking into account sampling weights and sampling design complexity in multiple imputed data. All covariates are measured in 2006 unless stated otherwise. ^b^Based on linear or multinomial logit models with sex or race as the only predictors of continuous and categorical variables, respectively, accounting for sampling weights and sampling design complexity in multiple imputed data. (Ref) is included to show which category (the one with the largest sample) was used as the referent in the multinomial logistic regression models. ^c^Number of chronic conditions among hypertension, diabetes, heart disease and stroke.

Figure 1 displays the likelihood of survival based on sleep quality tertiles and dementia level, as calculated by the 3 algorithms. In general, there was an association detected between low sleep quality and an increased death rate, and a clear link was seen where the mortality rate increased with the severity of poor sleep quality. Furthermore, a robust association was found between the likely presence of dementia and mortality risk.

Table 2 presents Cox proportional hazards (PH) models, where the primary variable of interest is the continuous score for “poor sleep quality,” and the outcome is the risk of mortality. The table displays the adjusted Log_e_ of the hazard ratio (Loge (HR)) along with associated standard errors (SEs), based on multiple imputed data. The Cox proportional hazards models showed that each unit increase in the “poor sleep quality” score was linked with a hazard ratio (HR) of 1.02 (95% confidence interval: 1.01–1.04), with a *p*-value of less than 0.001. The analysis was adjusted for age, sex, and race/ethnicity. The observed association was exclusively found in older adult males and among older adults of White ethnicity, with a marked interaction effect based on race. However, after accounting for other characteristics such as socio-economic status, lifestyle, and health-related factors, the poor sleep score was found to have a negative relationship with the risk of mortality. The estimated hazard ratio (HR) was 0.97 (0.96–0.98) per unit increase, indicating a decrease in risk. This relationship was statistically significant with a *p*-value of less than 0.001. In the analysis where sleep quality tertile is included as the main factor (Analysis B), each increase in tertile of the “poor sleep quality” measure was found to be related with a 7% increase in the risk of mortality in the entire population, according to the reduced model. In the fully adjusted model, each tertile increase in the “poor sleep quality” score was shown to be associated with a 7% decrease in the risk of mortality. This association was mostly observed among older adult females and individuals from the Black/Hispanic/Others racial groupings. The statistical analysis showed significant interactions with sex and race (*P* < 0.05). Hence, the correlation between inadequate sleep quality and the risk of mortality is intricate and varies among different models.

**Table 2:** Poor sleep quality, dementia odds (Log_e_ transformed) and all-cause mortality: Cox PH models, overall, by sex and by race, HRS 2006–2020^a,e^.

	**Overall**	**Male**	**Female**	**White**	**Black/Hispanic/Others**	**P_sex_**	**P_race_**
	**β ± SE**	**β ± SE**	**β ± SE**	**β ± SE**	**β ± SE**		
Unweighted N	*N* = 6,718	*N* = 2,854	*N* = 3,864	*N* = 5,440	*N* = 1,278		
***Analysis A***							
**Reduced models**							
***Model 1A: Poor sleep quality***	**+0.022 ± 0.007^c^**	**+0.030 ± 0.010^c^**	+0.016 ± 0.009	**+0.026 ± 0.007^d^**	−0.009 ± 0.015	0.27	**0.021**
***Model 1B: Hurd dementia***	**+0.085 ± 0.010^d^**	**+0.103 ± 0.005^d^**	**+0.082 ± 0.011^d^**	**+0.086 ± 0.011^d^**	**+0.082 ± 0.018^d^**	0.17	0.59
***Model 1C: Expert dementia***	**+0.151 ± 0.009^d^**	**+0.144 ± 0.005^d^**	**+0.156 ± 0.010^d^**	**+0.156 ± 0.009^d^**	**+0.114 ± 0.014^d^**	0.69	**0.001**
***Model 1D: LASSO dementia***	**+0.194 ± 0.011^d^**	**+0.180 ± 0.014^d^**	**+0.207 ± 0.014 ^d^**	**+0.197 ± 0.011^d^**	**+0.174 ± 0.018^d^**	0.84	**0.028**
**Full models**	*N* = 6,368	*N* = 2,653	*N* = 3,715	*N* = 5,204	*N* = 1,164		
***Model 2A: Poor sleep quality***	**−0.033 ± 0.009^c^**	**−0.016 ± 0.013**	**−0.044 ± 0.012^c^**	**−0.030 ± 0.010^c^**	**−0.057 ± 0.022^c^**	*0.099*	**0.032**
***Model 2B: Hurd dementia***	**+0.098 ± 0.010^d^**	**+0.080 ± 0.015^d^**	**+0.114 ± 0.012^d^**	**+0.100 ± 0.011^d^**	**+0.084 ± 0.017^d^**	0.99	**0.041**
***Model 2C: Expert dementia***	**+0.101 ± 0.009^d^**	**+0.075 ± 0.013^d^**	**+0.118 ± 0.011^d^**	**+0.108 ± 0.009^d^**	**+0.065 ± 0.016^d^**	0.56	**0.002**
***Model 2D: LASSO dementia***	**+0.150 ± 0.013^d^**	**+0.121 ± 0.018^d^**	**+0.172 ± 0.016^d^**	**+0.153 ± 0.014^d^**	**+0.125 ± 0.024^d^**	0.74	**0.048**
***Analysis B***							
**Reduced models**	*N* = 6,718	*N* = 2,854	*N* = 3,864	*N* = 5,440	*N* = 1,278		
***Model 1A: Poor sleep quality***	**+0.072 ± 0.023^c^**	**+0.099 ± 0.034^c^**	**+0.052 ± 0.028**	**+0.088 ± 0.023^d^**	−0.026 ± 0.048	0.26	**0.017**
***Model 1B: Hurd dementia***	**+0.675 ± 0.057^d^**	**+0.674 ± 0.080^d^**	**+0.684 ± 0.081^d^**	**+0.706 ± 0.058^d^**	**+0.563 ± 0.116^d^**	0.73	**0.012**
***Model 1C: Expert dementia***	**+0.731 ± 0.055^d^**	**+0.716 ± 0.063^d^**	**+0.743 ± 0.078^d^**	**+0.745 ± 0.062^d^**	**+0.673 ± 0.095^d^**	0.90	0.16
***Model 1D: LASSO dementia***	**+0.681 ± 0.058^d^**	**+0.708 ± 0.069^d^**	**+0.679 ± 0.083^d^**	**+0.696 ± 0.064^d^**	**+0.658 ± 0.092^d^**	0.36	*0.069*
**Full models**	*N* = 6,368	*N* = 2,653	*N* = 3,715	*N* = 5,204	*N* = 1,164		
***Model 2A: Poor sleep quality***	**−0.069 ± 0.027^b^**	−0.011 ± 0.037	**−0.110 ± 0.033^c^**	−0.056 ± 0.030	**−0.147 ± 0.067^b^**	**0.036**	**0.037**
***Model 2B: Hurd dementia***	**+0.400 ± 0.065^d^**	**+0.304 ± 0.088^d^**	**+0.461 ± 0.088^d^**	**+0.428 ± 0.070^d^**	**+0.300 ± 0.117^b^**	0.78	*0.055*
***Model 2C: Expert dementia***	**+0.457 ± 0.057^d^**	**+0.369 ± 0.069^d^**	**+0.510 ± 0.089^d^**	**+0.474 ± 0.066^d^**	**+0.422 ± 0.096^d^**	0.80	0.13
***Model 2D: LASSO dementia***	**+0.415 ± 0.067^d^**	**+0.448 ± 0.073^d^**	**+0.410 ± 0.097^d^**	**+0.416 ± 0.077^d^**	**+0.437 ± 0.102^d^**	0.26	0.37

Abbreviations: HRS: Health and Retirement Study; PH: Proportional hazards; SE: Standard Error. ^a^Values are β ± SE, with β representing Log_e_HR from Cox PH model for each exposure-outcome relationship. Cox PH models were conducted overall and across sex or race/ethnicity groups, taking into account sampling weights and sampling design complexity in multiple imputed data. All covariates are measured in 2006 unless stated otherwise. Analysis A included continuous forms of dementia probability (Log_e_ (odds of dementia)) and continuous “poor sleep quality” score; while Analysis B included binary dementia status (3 algorithms) and “poor sleep quality tertile”. ^b^*P* < 0.05; ^c^*P* < 0.010; ^d^*P* < 0.001 for null hypothesis that β = 0. ^e^Reduced models (Models 1A–1D) were adjusted for age at 2006, sex and race/ethnicity. Full models (Model 2A-2D) further adjusted the reduced model by all covariates described under the Covariates section, including socio-demographic, socio-economic, lifestyle and health-related factors.

Table 2 also incorporates three distinct algorithms to calculate the likelihood of dementia. These estimates were subsequently inputted into a Cox PH model to assess the risk of all-cause mortality. The modeling approach used for the “poor sleep quality” exposures was identical. The likelihood of developing dementia (after Log_e_ transformation) was consistently linked to an increased risk of mortality, with the LASSO algorithm (reduced model) showing a maximum risk increase of 21%. Controlling for the other factors reduced the strength of the relationship between the Ln(odds) of dementia and the risk of mortality for most algorithms, but the relationship still remained statistically significant. Racial disparities were found in the relationship between dementia and mortality in the comprehensive models. Among White adults, the association was more pronounced (e.g., HR = 1.17 for LASSO algorithm, *p* < 0.001) compared to Black/Hispanic/Others adults (HR = 1.13 for LASSO algorithm, *p* < 0.001), for each unit increase in the Loge (odds of dementia). It should be noted that this increase corresponds to the probability of dementia moving from 0% to 73%. The study found that individuals with a probable dementia diagnosis, as determined by each algorithm, had a roughly two-fold higher risk of all-cause mortality in the reduced model. This association was still statistically significant in the full model, although the magnitude of the risk was reduced to approximately HR = 1.5. There were no discernible differences based on sex or race.

Through a series of sensitivity studies conducted on the data shown in Table 2 (available at https://github.com/baydounm/HRS_SLEEP_DEMENTIA_MORTALITY_SUPPLEMENT), additional adjustments were made to the models by including alcohol as one of the factors. Despite these adjustments, the relationship between sleep quality and mortality did not undergo significant changes. Furthermore, according to a separate sensitivity analysis, removing the covariates that were already included in the entire model by backward exclusion indicated that the most significant possible confounding factors were self-rated health, the CES-D score, and cardiometabolic disorders. However, none of these characteristics alone were capable of counteracting the association between sleep quality and the risk of mortality. Ultimately, when the alternative measure of sleep quality was used, excluding the item on sleep drugs, the results were comparable to those obtained from the main sleep quality measure in this specific analysis.

Table 3 displays results from Cox PH models for the association between dementia odds (Log_e_ transformed) and mortality risk, stratifying by “poor sleep quality” score tertile. In the fully adjusted models, as well as the reduced models for the Expert and LASSO algorithms, dementia odds had a stronger association with mortality risk in the two lower tertiles of “poor sleep quality” score, and a markedly weaker association in the uppermost tertile. In the full model, a dose-response relationship was found for all 3 algorithms (*P* < 0.001 for “poor sleep quality” score tertile by dementia odds (Log_e_ transformed) interaction).

**Table 3:** Dementia odds (Log_e_ transformed) and all-cause mortality across sleep quality tertiles: Cox PH models, HRS 2006–2020^a^.

	**T1**	**T2**	**T3**	**P_sleep_^f^**
	**β ± SE**	**β ± SE**	**β ± SE**	
***Analysis A***				
Unweighted *N*	*N* = 2,286	*N* =3,055	*N* =1,377	
**Reduced models^e^**				
***Model 1A: Hurd dementia***	**+0.082 ± 0.012^d^**	**+0.090 ± 0.018^d^**	**+0.080 ± 0.015^d^**	0.37
***Model 1B: Expert dementia***	**+0.158 ± 0.016^d^**	**+0.153 ± 0.010^d^**	**+0.132 ± 0.016^d^**	**0.018**
***Model 1C: LASSO dementia***	**+0.204 ± 0.018^d^**	**+0.200 ± 0.015^d^**	**+0.164 ± 0.019^d^**	**0.009**
**Full models^e^**	*N* =2,146	*N* =2,930	*N* =1,292	
***Model 2A: Hurd dementia***	**+0.119 ± 0.016^d^**	**+0.097 ± 0.013^d^**	**+0.066 ± 0.019^c^**	**<0.001**
***Model 2B: Expert dementia***	**+0.122 ± 0.015^d^**	**+0.093 ± 0.012^d^**	**+0.081 ± 0.019^d^**	**<0.001**
***Model 2C: LASSO dementia***	**+0.173 ± 0.020^d^**	**+0.144 ± 0.017^d^**	**+0.122 ± 0.028^d^**	**<0.001**
***Analysis B***				
Unweighted *N*	*N* =2,286	*N* =3,055	*N* =1,377	
**Reduced models ^e^**				
***Model 1A: Hurd dementia***	**+0.801 ± 0.101^d^**	**+0.673 ± 0.068^d^**	**+0.505 ± 0.117^d^**	**0.003**
***Model 1B: Expert dementia***	**+0.836 ± 0.094^d^**	**+0.746 ± 0.081^d^**	**+0.557 ± 0.107^d^**	**0.003**
***Model 1C: LASSO dementia***	**+0.717 ± 0.097^d^**	**+0.767 ± 0.083^d^**	**+0.479 ± 0.103^d^**	**0.014**
**Full models^e^**				
***Model 2A: Hurd dementia***	**+0.574 ± 0.097^d^**	**+0.397 ± 0.070^d^**	+0.195 ± 0.136	**0.001**
***Model 2B: Expert dementia***	**+0.649 ± 0.077^d^**	**+0.409 ± 0.097^d^**	**+0.291 ± 0.123^b^**	**0.001**
***Model 2C: LASSO dementia***	**+0.559 ± 0.086^d^**	**+0.483 ± 0.081^d^**	+0.136 ± 0.130	**0.001**

Abbreviations: HRS: Health and Retirement Study; PH: Proportional hazards; SE: Standard Error. ^a^Values are β ± SE, with β representing Log_e_ (HR) from Cox PH model for each exposure-outcome relationship. Cox PH models were conducted overall and across sex or race/ethnicity groups, accounting for sampling weights and sampling design complexity in multiple imputed data. All covariates are measured in 2006 unless stated otherwise. Analysis A included continuous forms of dementia probability (Log_e_ (odds of dementia)); while Analysis B included binary dementia status (3 algorithms). T1 corresponded to the first tertile of the “poor sleep quality” score (Mean ± SD: 0.49 ± 0.50, range: 0–1); T2 corresponded to the second tertile: (Mean ± SD: 2.90 ± 0.81, range: 2–4); T3 corresponded to the third tertile (Mean ± SD: 6.13±1.19, range: 5–9). ^b^*P* < 0.05; ^c^*P* < 0.010; ^d^*P* < 0.001 for null hypothesis that β = 0. ^e^Reduced models (Models 1A–1C) were adjusted for age at 2006, sex and race/ethnicity. Full models (Model 2A–2C) further adjusted the reduced model by all covariates described under the Covariates section, including socio-demographic, socio-economic, lifestyle and health-related factors. ^f^*P*-value associated with 2-way interaction term between poor sleep quality tertile and the main exposure dementia odds (Log_e_ transformed), in a model not stratified by “poor sleep quality” tertile.

Four-way decomposition of poor sleep quality’s total effect on mortality risk through dementia odds (Log_e_ transformed) is shown in Table 4. This modeling approach alternated between a reduced and a fully adjusted model and for all 3 algorithms. When Hurd dementia was considered, in the reduced model, the positive total effect of poor sleep on mortality consisted mainly of a CDE. For Expert algorithmically defined dementia as the mediator/moderator, the total effect consisted of all 4 components, with an apparent antagonistic interaction. There was a similar pattern observed for the LASSO algorithm. In the fully adjusted model, poor sleep quality was inversely related to mortality risk, with a significant PIE explaining around 18–20% of the total effect for all 3 algorithms. Nevertheless, for all 3 algorithms, INTMED explained another 5–8% of the total effect, suggesting an antagonistic interaction between poor sleep quality and dementia in relation to mortality risk. When sex and race-specific findings were examined, PIE in the full model was mainly significant among female and White adults, as were INTREF and INTMED for all 3 algorithms. Among Black/Hispanic/Others adults, the inverse association of poor sleep quality with mortality risk in the full model was mostly a CDE.

**Table 4:** Poor sleep quality and all-cause mortality: four-way decomposition models by dementia odds (Log_e_ transformed), overall, by sex and by race, HRS 2006–2020^a,b^.

	**TE**		**CDE**		**INTREF**		**INTMED**		**PIE**	
**Y: All-cause mortality; X: Poor sleep quality score**	**β ± SE**	***P***	**β ± SE**	***P***	**β ± SE**	***P***	**β ± SE**	***P***	**β ± SE**	***P***
**Overall**										
**Reduced Model 1A–1C (*N* = 6,991)**										
M: Hurd	**+0.041 ± 0.015**	**0.007**	**+0.038 ± 0.015**	**0.010**	−0.001 ± 0.003	0.81	+0.0000 ± 0.0002	0.83	+0.0041 ± 0.0034	0.24
M: Expert	**+0.033 ± 0.015**	**0.035**	**0.031 ± 0.014**	**0.030**	**−0.014 ± 0.004**	**0.001**	**−0.002 ± 0.001**	**0.030**	**+0.017 ± 0.005**	**<0.001**
M: LASSO	**+0.032 ± 0.015**	**0.036**	**+0.036 ± 0.014**	**0.010**	**−0.011 ± 0.004**	**0.005**	−0.001 ± 0.000	0.19	***+0.008 ± 0.005***	***0.096***
**Full model 2A–2C; (*N* = 6,510)**										
M: Hurd	**−0.0654 ± 0.0161**	**<0.001**	**−0.06400 ± 0.01642**	**<0.001**	**+0.00553 ± 0.00175**	**0.002**	**+0.00363 ± 0.00114**	**0.001**	**−0.01054 ± 0.00295**	**<0.001**
M: Expert	**−0.0667 ± 0.0161**	**<0.001**	**−0.05895 ± 0.01636**	**<0.001**	+0.00079 ± 0.00135	**0.53**	**+0.00391 ± 0.00111**	**<0.001**	**−0.01249 ± 0.00304**	**<0.001**
M: LASSO	**−0.0684 ± 0.0161**	**<0.001**	**−0.05959 ± 0.01641**	**<0.001**	***+0.00226 ± 0.00139***	***0.094***	**+0.00442 ± 0.00116**	**<0.001**	**−0.01547 ± 0.00330**	**<0.001**
**Male**										
**Reduced Model 1A–1C (*N* = 2,931)**										
M: Hurd	***+0.04174 ± 0.02375***	***0.079***	***+0.04182 ± 0.02267***	***0.065***	**−0.01249 ± 0.00387**	**0.001**	***−0.00226 ± 0.00128***	***0.079***	**+0.01467 ± 0.00625**	**0.019**
M: Expert	***+0.04685 ± 0.02408***	***0.052***	+0.03150 ± 0.02261	0.16	−0.00721 ± 0.00462	0.12	−0.00170 ± 0.00153	0.26	**+0.02426 ± 0.00687**	**<0.001**
M: LASSO	***+0.04592 ± 0.02394***	***0.055***	+0.03582 ± 0.02277	0.12	**−0.00741 ± 0.00347**	**0.032**	−0.00165 ± 0.00125	0.19	**+0.01917 ± 0.00691**	**0.006**
**Full model 2A–2C (*N* = 2,698)**										
M: Hurd	***−0.04648 ± 0.02552***	***0.069***	***−0.05095 ± 0.02573***	**0.048**	**+0.00721 ± 0.0030**	**0.018**	+0.00173 ± 0.00141	0.22	−0.00447 ± 0.00348	0.20
M: Expert	***−0.04624 ± 0.02559***	***0.071***	***−0.04757 ± 0.02574***	***0.065***	**+0.00535 ± 0.00258**	**0.038**	***+0.00215 ± 0.00129***	***0.096***	−0.00447 ± 0.00348	0.20
M: LASSO	***−0.04828 ± 0.02547***	***0.058***	**−0.05299 ± 0.02632**	**0.044**	**+0.01054 ± 0.00426**	**0.013**	+0.00204 ± 0.00124	0.10	**−0.00786 ± 0.00398**	**0.048**
**Female**										
**Reduced Model 1A–1C (*N* = 4,060)**										
M: Hurd	***+0.03516 ± 0.02026***	***0.083***	+0.03029 ± 0.01894	0.11	+0.00402 ± 0.00469	0.39	+0.00005 ± 0.00027	0.86	+0.00081 ± 0.00459	0.86
M: Expert	+0.02303 ± 0.02030	0.26	+0.029170 ± 0.01832	0.11	**−0.01868 ± 0.00636**	0.003	−0.00147 ± 0.00091	0.11	**0.01401 ± 0.00678**	**0.039**
M: LASSO	+0.02335 ± 0.02028	0.25	***+0.03486 ± 0.01820***	***0.055***	***−0.01336 ± 0.00697***	***0.055***	−0.00013 ± 0.00044	0.76	+0.00198 ± 0.00644	0.76
**Full model 2A–C (*N* = 3,812)**										
M: Hurd	**−0.07924 ± 0.02084**	**<0.001**	**−0.07305 ± 0.02132**	**0.001**	***+0.00380 ± 0.00223***	***0.088***	**+0.00489 ± 0.00169**	**0.004**	**−0.01488 ± 0.00446**	**0.001**
M: Expert	**−0.08221 ± 0.02080**	**0.001**	**−0.06579 ± 0.02109**	**0.002**	**−0.00454 ± 0.00218**	**0.037**	**+0.00556 ± 0.00175**	**0.001**	**−0.01743 ± 0.00480**	**0.001**
M: LASSO	**−0.08393 ± 0.02072**	**<0.001**	**−0.06101 ± 0.02096**	**0.004**	**−0.00801 ± 0.00237**	**0.001**	**+0.00657 ± 0.00185**	**<0.001**	**−0.02148 ± 0.00499**	**<0.001**
**White**										
**Reduced Model 1A–1C (*N* = 5,666)**										
M: Hurd	**+0.05311 ± 0.01733**	**0.002**	**+0.05371 ± 0.01668**	**0.001**	−0.00020 ± 0.00257	0.94	−0.00001 ± 0.00013	0.92	−0.00039 ± 0.00383	0.92
M: Expert	**+0.04577 ± 0.01742**	**0.009**	**+0.04676 ± 0.01627**	**0.004**	**−0.00948 ± 0.00365**	**0.009**	−0.00055 ± 0.00047	0.25	***+0.00904 ± 0.00547***	***0.098***
M: LASSO	**+0.04335 ± 0.01728**	**0.012**	**+0.05197 ± 0.01625**	**0.001**	**−0.00933 ± 0.00367**	**0.011**	−0.00005 ± 0.00031	0.89	0.00075 ± 0.00523	0.89
**Full model 2A–2C (*N* = 5,324)**										
M: Hurd	**−0.04948 ± 0.01799**	**0.006**	**−0.05492 ± 0.01857**	**0.003**	**+0.01331 ± 0.00308**	**<0.001**	**+0.00411 ± 0.00132**	**0.002**	**−0.01197 ± 0.00339**	**<0.001**
M: Expert	**−0.05117 ± 0.01799**	**0.004**	**−0.04568 ± 0.01847**	**0.013**	**+0.00634 ± 0.00200**	**0.001**	**+0.00515 ± 0.00136**	**<0.001**	**−0.01698 ± 0.00366**	**<0.001**
M: LASSO	**−0.05237 ± 0.01791**	**0.003**	**−0.04696 ± 0.01853**	**0.011**	**+0.00781 ± 0.00224**	**<0.001**	**+0.00567 ± 0.00143**	**<0.001**	**−0.01888 ± 0.00384**	**<0.001**
**Black/Hispanic/Others**										
**Reduced Model 1A–1C (*N* = 1,325)**										
M: Hurd	−0.00793 ± 0.03387	0.82	−0.02466 ± 0.03107	0.43	−0.00388 ± 0.01139	0.73	−0.00068 ± 0.00175	0.70	0.02129 ± 0.00785	**0.007**
M: Expert	−0.01939 ± 0.03425	0.57	−0.02685 ± 0.03152	0.39	***−0.02899 ± 0.01722***	***0.092***	−0.00688 ± 0.00426	0.11	**+0.04332 ± 0.01079**	**<0.001**
M: LASSO	−0.01300 ± 0.03459	0.71	−0.02891 ± 0.03043	0.34	−0.01620 ± 0.01634	0.32	−0.00313 ± 0.00310	0.31	**+0.03524 ± 0.01061**	**0.001**
**Full model 2A–2C (*N* = 1,186)**										
M: Hurd	**−0.13082 ± 0.03743**	**<0.001**	**−0.11055 ± 0.03785**	**<0.001**	−0.01620 ± 0.01051	0.12	+0.00149 ± 0.00175	0.39	−0.00556 ± 0.00571	0.33
M: Expert	**−0.13009 ± 0.03742**	**0.001**	**−0.11917 ± 0.03778**	**0.002**	−0.01013 ± 0.00868	0.24	+0.00024 ± 0.00116	0.84	−0.00103 ± 0.00490	0.84
M: LASSO	**−0.13380 ± 0.03736**	**<0.001**	**−0.12390 ± 0.03678**	**0.001**	−0.00588 ± 0.00867	0.50	+0.00059 ± 0.00103	0.57	−0.00462 ± 0.00626	0.46

Abbreviations: CDE: Controlled Direct Effect; HRS: Health and Retirement Study; INTMED: Mediated Interaction; INTREF: Interaction Referent; PIE: Pure Indirect Effect; SE: Standard Error. ^a^Cox PH regression models with mortality as the main outcome and poor sleep quality as the exposure. Log_e_ (odds (dementia probability)) using Hurd, expert and LASSO algorithms were potential mediators/moderators allowed to interact with the main exposure, four-way decomposition analysis. 1 SD of the “poor sleep quality score” corresponded to 2.18 point higher score. 1 SD of Hurd, expert and LASSO algorithm Log_e_ (dementia probability) corresponded to 3.93, 3.13 and 2.40, respectively. ^b^Exogenous variables are the ones included in Table 2, Models 1A–1D and 2A–2D, as covariates for the reduced and full models, respectively. See Covariates section for detail.

Four-way decomposition of dementia odds (Log_e_ transformed) total effect on mortality risk through poor sleep quality is shown in Table 5. As shown before, attenuation of the total effect was observed between reduced and full models, and in both cases, dementia odds were directly associated with mortality risk. This total effect in the reduced model was largely due to a controlled direct effect. In the full model, around 5–8% of the total effect was due to INTREF or INTMED, while a smaller portion was due to PIE (~1–2%). These patterns were mostly observed among White participants, while among Black/Hispanic/Others individuals, the TE was mostly a CDE in the fully adjusted model. These patterns were detected for all 3 algorithms. In a sensitivity analysis whereby the sleep quality measure was replaced with one that excluded the sleep medication item, results were not markedly altered. The findings are depicted in Supplementary Tables 1 and 2 and the full output provided on https://github.com/baydounm/HRS_SLEEP_DEMENTIA_MORTALITY_SUPPLEMENT.

**Table 5:** Dementia odds (Log_e_ transformed) and all-cause mortality: four-way decomposition models by poor sleep quality, overall, by sex and by race: HRS 2006–2020^a,b^.

	**TE**		**CDE**		**INTREF**		**INTMED**		**PIE**	
**Y: All-cause mortality; M: Poor sleep quality score**	**β ± SE**	***P***	**β ± SE**	***P***	**β ± SE**	***P***	**β ± SE**	***P***	**β ± SE**	***P***
**Overall**										
**Reduced Model 1A–1C (*N* = 6,991)**										
X: Hurd	**+0.39525 ± 0.01906**	**<0.001**	**+0.39470 ± 0.01884**	**<0.001**	−0.00014 ± 0.00049	0.78	+0.00004 ± 0.00028	0.88	+0.00064 ± 0.00059	0.28
X: Expert	**0.62033 ± 0.02985**	**<0.001**	**+0.62222 ± 0.02984**	**<0.001**	−0.00023 ± 0.00121	0.85	**−0.00339 ± 0.00154**	**0.028**	***+0.00172 ± 0.00092***	***0.061***
X: LASSO	**0.58242 ± 0.02680**	**<0.001**	**+0.58335 ± 0.02676**	**<0.001**	−0.00070 ± 0.00089	0.43	−0.00115 ± 0.00086	0.18	+0.00092 ± 0.00065	0.16
**Full model 2A–2C (*N* = 6,510)**										
X: Hurd	**+0.50781 ± 0.04782**	**<0.001**	**+0.46837 ± 0.04583**	**<0.001**	**+0.01866 ± 0.00609**	**0.002**	**+0.01585 ± 0.00517**	**0.002**	**+0.00493 ± 0.00187**	**0.008**
X: Expert	**+0.38284 ± 0.03203**	**<0.001**	**+0.35900 ± 0.03112**	**<0.001**	**+0.01024 ± 0.00345**	**0.003**	**+0.00947 ± 0.00278**	**0.001**	**+0.00414 ± 0.00153**	**0.007**
X: LASSO	**+0.44570 ± 0.03455**	**<0.001**	**+0.41699 ± 0.03368**	**<0.001**	**+0.01151 ± 0.00389**	**0.003**	**+0.01224 ± 0.00335**	**<0.001**	**+0.00496 ± 0.00174**	**0.004**
**Male**										
**Reduced Model 1A–1C (*N* = 2,931)**										
X: Hurd	**+0.56784 ± 0.04557**	**<0.001**	**+0.55737 ± 0.04524**	**<0.001**	***+0.01378* ± 0*.00707***	***0.051***	***−0.00581* ± 0*.00328***	***0.076***	+0.00251 ± 0.00170	0.14
X: Expert	**+0.60686 ± 0.05046**	**<0.001**	**+0.60202 ± 0.05059**	**<0.001**	+0.00639 ± 0.00618	0.30	−0.00446 ± 0.00385	0.24	+0.00291 ± 0.00221	0.19
X: LASSO	**+0.57377 ± 0.04415**	**<0.001**	**+0.56788 ± 0.04412**	**<0.001**	+0.00730 ± 0.00583	0.21	−0.00373 ± 0.00274	0.17	+0.00233 ± 0.00167	0.16
**Full model 2A–2C (*N* = 2,698)**										
X: Hurd	**+0.41642 ± 0.07246**	**<0.001**	**+0.36637 ± 0.06879**	**<0.001**	**+0.03971 ± 0.01513**	**0.009**	+0.00806 ± 0.00666	0.23	+0.00228 ± 0.00210	0.28
X: Expert	**+0.26959 ± 0.04759**	**<0.001**	**+0.24091 ± 0.04630**	**<0.001**	**+0.02081 ± 0.00885**	**0.019**	+0.00534 ± 0.00328	0.10	+0.00254 ± 0.00189	0.18
X: LASSO	**.3520528 ± .0514405**	**<0.001**	**.3215936 .0500869 ±**	**<0.001**	**.0218936 ± .0099902**	**0.028**	.0057126 ± .003571	0.11	.0028529 ± .0020199	0.16
**Female**										
**Reduced Model 1A–1C (*N* = 4,060)**										
X: Hurd	**+0.37651 ± 0.02293**	**<0.001**	**+0.37352 ± 0.02191**	**<0.001**	+0.00283 ± 0.00294	0.34	+0.00007 ± 0.00039	0.86	+0.00009 ± 0.00054	0.86
X: Expert	**+0.63482 ± 0.03735**	**<0.001**	**+0.64365 ± 0.03736**	**<0.001**	**−0.00729 ± 0.00256**	**0.004**	−0.00278 ± 0.00172	0.11	+0.00123 ± 0.00096	0.20
X: LASSO	**+0.60279 ± 0.03461**	**<0.001**	**+0.60793 ± 0.03429**	**<0.001**	***−0.00511 ± 0.00281***	***0.068***	−0.00024 ± 0.00080	0.76	+0.00022 ± 0.00071	0.76
**Full model 2A–2C (*N* = 3,812)**										
X: Hurd	**+0.56909 ± 0.06359**	**<0.001**	**+0.54232 ± 0.06188**	**<0.001**	−0.00075 ± 0.00514	0.88	**+0.02080 ± 0.00754**	**0.006**	**+0.00673 ± 0.00282**	**0.017**
X: Expert	**+0.46129 ± 0.04325**	**<0.001**	**±0.44589 ± 0.04242**	**<0.001**	−0.00320 ± 0.00340	0.35	**+0.01338 ± 0.00440**	**0.002**	**+0.00522 ± 0.00223**	**0.019**
X=LASSO	**+0.51349 ± 0.04663**	**<0.001**	**+0.49309 ± 0.04586**	**<0.001**	−0.00386 ± 0.00357	0.28	**+0.01806 ± 0.00535**	**0.001**	**+0.00619 ± 0.00260**	**0.017**
**White**										
**Reduced Model 1A–1C (*N* = 5,666)**										
X: Hurd	**+0.40191 ± 0.02179**	**<0.001**	**+0.40169 ± 0.02137**	**<0.001**	+0.00032 ± 0.00128	0.81	−0.00002 ± 0.00019	0.91	−0.00008 ± 0.00083	0.92
X: Expert	**+0.65884 ± 0.03420**	**<0.001**	**+0.66085 ± 0.03412**	**<0.001**	***−0.00201 ± 0.00104***	***0.053***	−0.00132 ± 0.00109	0.23	+0.00132 ± 0.00091	0.15
X: LASSO	**+0.59003 ± 0.02964**	**<0.001**	**+0.59204 ± 0.02956**	**<0.001**	**−0.00203 ± 0.00100**	**0.042**	−0.00009 ± 0.00065	0.89	+0.00012 ± 0.00083	0.89
**Full model 2A–2C (*N* = 5,324)**										
X: Hurd	**+0.52716 ± 0.05367**	**<0.001**	**+0.48794 ± 0.05143**	**<0.001**	**+0.01634 ± 0.00657**	**0.013**	**+0.01836 ± 0.00614**	**0.003**	**+0.00452 ± 0.00200**	**0.043**
X: Expert	**+0.43335 ± 0.03742**	**<0.001**	**+0.40639 ± 0.03639**	**<0.001**	**+0.00910 ± 0.00388**	**0.019**	**±0.01379 ± 0.00380**	**<0.001**	**+0.00406 ± 0.00188**	**0.030**
X: LASSO	**+0.47401 ± 0.03910**	**<0.001**	**+0.44245 ± 0.03806**	**<0.001**	**+0.01079 ± 0.00438**	**0.014**	**+0.01629 ± 0.00431**	**<0.001**	**+0.00448 ± 0.00201**	**0.026**
**Black/Hispanic/Others**										
**Reduced Model 1A–1C (*N* = 1,325)**										
X: Hurd	**+0.36174 ± 0.03989**	**<0.001**	**+0.36399 ± 0.04016**	**<0.001**	+0.00134 ± 0.00387	0.73	−0.00105 ± 0.00274	0.70	−0.00254 ± 0.00336	0.45
X: Expert	**+0.47987 ± 0.06066**	**<0.001**	**+0.48313 ± 0.06060**	**<0.001**	+0.01265 ± 0.00903	0.16	−0.01126 ± 0.00714	0.12	−0.00465 ± 0.00564	0.41
X: LASSO	**+0.53338 ± 0.06233**	**<0.001**	**+0.53659 ± 0.06229**	**<0.001**	+0.00674 ± 0.00757	0.37	−0.00578 ± 0.00582	0.32	−0.00417 ± 0.00461	0.37
**Full model 2A–2C (*N* = 1,186)**										
X: Hurd	**+0.41028 ± 0.10579**	**<0.001**	**+0.37851 ± 0.10205**	**<0.001**	+0.01963 ± 0.01694	0.25	+0.00605 ± 0.00738	0.41	+0.00608 ± 0.00652	0.35
X: Expert	**±0.22589 ± 0.06172**	**<0.001**	**+0.21525 ± 0.05966**	**<0.001**	+0.00927 ± 0.00966	0.34	+0.00042 ± 0.00205	0.84	+0.00095 ± 0.00453	0.83
X: LASSO	**+0.33939 ± 0.07511**	**<0.001**	**+0.32810 ± 0.07360**	**<0.001**	+0.00591 ± 0.01086	0.59	+0.00133 ± 0.00252	0.60	+0.00405 ± 0.00560	0.47

Abbreviations: CDE: Controlled Direct Effect; HRS: Health and Retirement Study; INTMED: Mediated Interaction; INTREF: Interaction Referent; PIE: Pure Indirect Effect; SE: Standard Error. ^a^Cox PH regression models with mortality as the main outcome and poor sleep quality as the potential mediator/moderator allowed to interact with the main exposure. Log_e_ (odds (dementia probability)) using Hurd, expert and LASSO algorithms were the main alternative exposures of interest, sample size *N* = 6,991, four-way decomposition analysis. 1 SD of the “poor sleep quality score” corresponded to 2.18 point higher score. 1 SD of Hurd, expert and LASSO algorithm Log_e_ (dementia probability) corresponded to 3.93, 3.13 and 2.40, respectively. ^b^Exogenous variables are the ones included in Table 2, Models 1A–1D and 2A–2D, as covariates for the reduced and full models, respectively. See Covariates section for detail.

Furthermore, when each item was tested separately in relation to all-cause mortality and dementia status, some items were more predictive of each of these two outcomes than others. More specifically, there was a dose-response relationship between poor sleep based on “trouble falling asleep” and all-cause mortality (Poor vs. Good: HR = 1.17, *P* < 0.001; Medium vs. Good: HR = 0.95, *P* = 0.10), as was the case for “feeling rested in the morning” (Poor vs. Good: HR = 1.27, *P* < 0.001; Medium vs. Good: HR = 1.16, *P* < 0.001). Similarly, medication use for sleep was associated with increased risk for all-cause mortality (HR = 1.13, *P* < 0.001). In contrast, “trouble waking up during the night” was inversely related to dementia status based on all 3 algorithms unlike “trouble falling asleep” was, as expected, associated with greater risk for dementia particularly when comparing “Poor” with “Good” sleep quality. “Feeling rested in the morning” (“Poor vs. Good”, and “Medium vs. Good”) was associated with greater risk for dementia, for all 3 algorithms. No associated was detected for the remaining items including medication use for sleep, with respect to dementia status. The full output for this sensitivity analysis can also be found on: https://github.com/baydounm/HRS_SLEEP_DEMENTIA_MORTALITY_SUPPLEMENT.

## DISCUSSION

### Summary of findings

Our present study examines the bi-directional associations of sleep quality, dementia status and mortality in older adults residing in the US, by utilizing data from the Health and Retirement Study. It found that poor sleep quality was associated with increased all-cause mortality risk in males and among White older adults. The association was strongest among White adults, but attenuated in fully adjusted models. The study also detected a stronger positive association between dementia and mortality among individuals with better sleep quality. Four-way decomposition indicated roles played by both mediation and interaction, though statistically significant total effects were mainly composed of controlled direct effects.

### Previous studies

#### Sleep and mortality

The current study’s findings of an association between sleep and mortality, particularly within males, aligns with some of these previous literature [38]. Specifically, one study observed insomnia and excessive daytime sleepiness strictly within older men was associated with an increased risk of mortality over 20 years even after accounting for other health conditions (e.g., cancer, depression, dementia) [38]. However, this observation is not entirely consistent with the prior evidence. For example, some prior studies have not observed a significant association between sleep and mortality risk but have observed an association between sleep and health outcomes (e.g., stroke, coronary heart disease) that increase mortality risk [39, 40]. Inconsistencies in the current work and prior findings can be attributed to differences in the age ranges of the included participants, sleep measures, and approach for measuring mortality. Despite the differences, our findings warrant further exploration of biopsychosocial factors that might influence the sleep and mortality association particularly within men.

There is a connection between sleep disturbance and increased systemic inflammation, which can cause damage to neurons and result in death [3, 41]. Research has investigated the correlation between sleep and several health conditions such as obesity, diabetes, heart disease, depression, cholesterol, and inflammation [3, 42]. We cannot make definitive judgments on the relationship between sleep quality and disorders and their ability to predict all-cause mortality due to the lack of sufficient evidence. A detailed review of prior studies is provided elsewhere [3]. Similarly, a study examining sleep behaviors and all-cause mortality rates in older adults using data from the NHANES, found a positive relationship between long sleep and mortality rates [3]. Sleepiness/disorder was positively associated with mortality rates in males, while poor sleep-related daytime dysfunction negatively affected mortality rates in elderly individuals [3]. In our current investigation, while the exact duration of sleep was not evaluated, certain indicators of sleep quality were found to be more prognostic of overall mortality compared to others. These indicators include the use of medicine, difficulty in initiating sleep, and feeling unrefreshed in the morning. These findings require additional replication in other nationally representative cohorts, specifically among older persons.

#### Dementia and mortality

Impaired cognitive function assessed in the middle to late stages of life is associated with a higher likelihood of mortality, although the specific reasons for this relationship remain unclear. Cognitive function is primarily influenced by intricate interplay between environmental and genetic factors over the course of one’s life, which can also impact health and lifespan [43, 44]. Deviation from normal cognitive performance may indicate underlying biological problems or hereditary variables that extend beyond neurodegenerative illnesses, as well as social and physical consequences [18, 45]. Significantly, a comprehensive analysis of over 60 research studies revealed that cognitive impairment, including obvious dementia, was linked to a higher likelihood of death from any cause [18, 46]. In contrast to previous investigations, the present study found that the link between dementia and mortality is most pronounced among White adults. This finding is consistent with a previous meta-analysis that examined multiple studies [46]. This observation is thought to be caused by methodological difficulties, such as the lack of representation of certain racial or ethnic groups who may not have access to health services for a dementia diagnosis. It could also be influenced by sociohistorical events or experiences, such as migration patterns, that are especially relevant to the lifespan of specific racial or ethnic groups [47]. Nevertheless, it is necessary to do further research in order to properly examine these potential causes.

#### Sleep and dementia

Recent research has found that both insufficient sleep (less than 6 hours) and excessive sleep (more than 9 hours) are linked to impaired cognitive performance and higher chances of Alzheimer’s disease and dementias [15, 48]. Gildner and colleagues uncovered an association between prolonged sleep duration and decreases in total cognitive function, attention/working memory, and executive function [12, 15]. These findings have been corroborated with others as described in another recent study [15]. Importantly, the latter study involving 9518 US elderly participants in the HRS, found that severe insomnia symptoms increased the risk of memory problems and dementia diagnoses over a 10-year follow-up period [15]. According to a comprehensive meta-analysis, persons who reported having trouble sleeping had a higher risk of developing dementia, AD, and cerebrovascular disease [49]. Furthermore, there is a correlation between sleep disturbances, including sleep disordered breathing, and an increased occurrence of dementia, specifically AD and cerebrovascular disease [49]. Prior studies have also proposed that sleep disturbances could serve as an early indicator of cognitive dysfunction (e.g., [16]), or may share common risk factors, or conversely, may be associated with a greater risk of cognitive dysfunction or mortality due to medication usage. Therefore, further comprehensive investigations are necessary.

### Interaction between sleep and cognition in relation to mortality risk

Additional research has shown a relationship between the risk of death and cognition. For example, self-reported short sleep duration (HR: 1.03 (0.98–1.09)) and long sleep duration (HR: 1.13 (1.08–1.18)) were linked to higher risk of mortality, according to data from the Chinese Longitudinal Healthy Longevity Surveys [50]. In a stratified study based on physical disability, chronic diseases, and cognitive impairment, only those with MMSE scores ≤24 had a mortality risk (60). Long sleep and cognitive impairment had a statistically significant relationship with mortality (P for interaction = .002) [50]. A population-based, in-lab, longitudinal study from the Penn State Adult Cohort revealed that those who slept less than 6 hours at baseline had a significantly higher risk of mortality from all causes and mortality linked to potential vascular cognitive impairment (*n* = 122) (HR = 1.79, 95% confidence interval (CI) = 1.28–2.51 and HR = 4.01, 95% CI = 2.66–6.05, respectively) [51]. Both of these studies suggest some synergism between poor sleep and poor cognition in relation to mortality risk. Those findings are in contrast with our present study, the latter showing that the association between dementia probability and mortality risk was mostly detected among individuals with better sleep quality. Thus, more studies are needed in order to come to a consensus with respect to interactions between sleep and cognition in relation to mortality risk, and whether these interactions differ across different age groups.

### Heterogeneity by race

In previous studies, compared to White adults, Black adults tend to report short (<7 hours) or long (>10 hours) durations of sleep, trouble falling asleep, worse sleep efficiency, and greater daytime sleepiness [52]. Moreover, sleep disorders (e.g., sleep apnea) tend to be highly prevalent among Black individuals [53]. Despite the disproportionate risk of dementia [54] and mortality [55] observed in Black adults, we found that dementia was more strongly associated with mortality among White adults. However, the Case and Deaton (2017) paper illustrates that mortality rates increased in 2015 particularly for non-Hispanic White adults with lower education levels while mortality rates declined/remained stable for Black and Hispanic individuals during this time frame [55]. This observation brings up the possibility that the dementia and mortality association may be particularly strong among White adults with lower education levels. This might speak to further exploration of how socioeconomic heterogeneity among White adults might further elucidate the group of individuals that may be at risk. Other literature is reviewed in the Supplementary Materials under “Supplementary literature search and reviews”.

### Strengths and limitations

Our study has notable strengths. First, this is the first investigation to test the association of sleep and dementia with mortality risk, while also exploring bi-directional mediational effects. It is also the first to accomplish this by using a nationally representative study of older adults who were followed for up to 14 years [18]. The HRS contains a wealth of data, allowing us to test numerous hypotheses while accounting for the potential confounding effects of extraneous variables. The present study also made use of advanced techniques, including multiple imputations applied to covariates, survival methods such as Cox proportional hazards models accounting for sampling design complexity and four-way decomposition models to test both mediation and interaction simultaneously [18]. Nevertheless, our study has some notable limitations. First, although date of death was available, date of birth was only an estimate at the month and year precision level, adding some measurement error into the analysis [18]. Second, poor sleep quality was measured using a minimal set of questions that were not validated against other measures such as the full PSQI score or more objective measures such as those obtained from an accelerometer. Third, measurement error in the algorithmically defined dementia outcome could not be assessed, although some of these algorithms relied heavily on the extensive ADAMS sub-study [18]. Finally, we cannot rule out the role played by residual confounding and selection biases.

## CONCLUSIONS

In sum, poor sleep quality was only directly related to mortality risk before adjustment for lifestyle and health-related factors. Therefore, the potential causal effect of poor sleep quality on mortality risk appears to be confounding by other lifestyle and health-related factors. Dementia was positively associated with mortality risk, particularly among individuals with better sleep quality, among males and among White older adults. Furthermore, individuals with normal sleep quality should be screened for cognitive performance over time as it may be a predictor for adverse future health outcomes, particularly men and White older adults. Future studies should uncover some of the underlying mechanisms behind the dementia-sleep antagonistic interactions.

## Supplementary Materials

Supplementary Methods

Supplementary Figure

Supplementary Tables
