# Supplementary material for: Poor sleep quality, dementia status and their association with all-cause mortality among older US adults
Source: Aging (Albany NY). 2024 Sep 4;16(17):12138–67. doi: 10.18632/aging.206102 (PMC11424588; doi:10.18632/aging.206102)
Supplement: Supplementary Methods [file aging-16-206102-s001.pdf]

SUPPLEMENTARY METHODS

Supplementary Method 1: Algorithmically defined dementia outcomes

The algorithms estimate a predicted dementia probability using different combinations of socio-demographic characteristics, health and physical functioning variables, social engagement indicators, and cognitive indicators (i.e., cognition test item scores and proxy-reports of cognition) to classify dementia status using race/ethnicity-specific probability thresholds. Each algorithm was designed to minimize differences in prediction accuracy across race/ethnicity groups, with pairwise differences of 3 percentage points for sensitivity and 5 percentage points for specificity, and is thus suitable for use in race/ethnicity disparities research.

This data file (hrsdementia_20211109.sas7bdat) was created using the 2018 RAND V1 HRS longitudinal file ("randhrs1992_2018v1") and core HRS data; code for reproducing this dataset is available in the following Github repository, and is dated 2021_1109.

Note the small differences in the probabilities and classifications for all years in this dataset compared to the previously distributed dataset (hrsdementia_20191028.sas7bdat) due to differences in the source data. This previously distributed dataset covered 2000 to 2014, and was created using the 2014 RAND HRS longitudinal V2 file ("randhrs1992_2014v2") and core HRS data; code for reproducing this prior version of the dataset is available in the following Github repository and is dated 2019_0529: https://github.com/powerepilab/AD_algorithm_development.

Supplementary Method 2: Poor sleep quality

https://hrs.isr.umich.edu/sites/default/files/meta/2006/core/codebook/h06c_ri.htm

KC083 TROUBLE FALLING ASLEEP

Section: C Level: Respondent Type: Numeric Width: 1 Decimals: 0

*Ref: SecC.Sleep.C083_*

How often do you have trouble falling asleep?

Would you say most of the time, sometimes, or rarely or never?

....

2570	*1. MOST OF THE TIME*
5482	*2. SOMETIMES*
10382	*3. RARELY OR NEVER*
32	*8. DK (Don't Know); NA (Not Ascertained)*
1	*9. RF (Refused)*
2	*Blank. INAP (Inapplicable); Partial Interview*

KC084 TROUBLE WAKING UP DURING NIGHT

Section: C Level: Respondent Type: Numeric Width: 1 Decimals: 0

*Ref: SecC.Sleep.C084_*

How often do you have trouble with waking up during the night?

(Would you say most of the time, sometimes, or rarely or never?)

..

4783 1. MOST OF THE TIME

6257 2. SOMETIMES

7351 3. RARELY OR NEVER

71 8. DK (Don't Know); NA (Not Ascertained)

5 9. RF (Refused)

2 Blank. INAP (Inapplicable); Partial Interview

KC085 TROUBLE WAKING UP TOO EARLY

Section: C Level: Respondent Type: Numeric Width: 1 Decimals: 0

*Ref: SecC.Sleep.C085_*

How often do you have trouble with waking up too early and not being able tofall asleep again?

(Would you say most of the time, sometimes, or rarely or never?)

..

2535 1. MOST OF THE TIME

5349 2. SOMETIMES

10490 3. RARELY OR NEVER

90 8. DK (Don't Know); NA (Not Ascertained)

3 9. RF (Refused)

2 Blank. INAP (Inapplicable); Partial Interview

KC086 FEEL RESTED IN MORNING

Section: C Level: Respondent Type: Numeric Width: 1 Decimals: 0

*Ref: SecC.Sleep.C086_*

How often do you feel really rested when you wake up in the morning?

(Would you say most of the time, sometimes, or rarely or never?)

..

10955 1. MOST OF THE TIME

4482 2. SOMETIMES

2901 3. RARELY OR NEVER

127 8. DK (Don't Know); NA (Not Ascertained)

2 9. RF (Refused)

2 Blank. INAP (Inapplicable); Partial Interview

KC232U2 MEDICATIONS TO SLEEP

Section: C Level: Respondent Type: Numeric Width: 1 Decimals: 0

*Ref: SecC.Sleep.C232_*

In the past two weeks, have you taken any medications or used other treatmentsto help you sleep?

...

3337	*1. YES*
15077	*5. NO*
49	*8. DK (Don't Know); NA (Not Ascertained)*
4	*9. RF (Refused)*
2	*Blank. INAP (Inapplicable); Partial Interview*

ASK:

IF C232_ = YES

- -

KC233 MEDICATIONS RECOMMENDED BY DOCTOR

Section: C Level: Respondent Type: Numeric Width: 1 Decimals: 0

Ref: SecC.Sleep.C233_MEDICATIONS

Were these medications or other treatments recommended to you by a doctor?

...

2331	*1. YES*
1002	*5. NO*
4	*8. DK (Don't Know); NA (Not Ascertained)*
	9. RF (Refused)
15132	*Blank. INAP (Inapplicable); Partial Interview*

Supplementary Method 3: Four-way decomposition

In this study, each potential mediator/moderator was entered into four-way decomposition models, thus breaking down the relationship between a specific exposure and the common outcome (all-cause mortality risk) into four distinct parts: (i) neither mediation nor interaction, or controlled direct effect (CDE); (ii) interaction alone (and not mediation), or Interaction Referent (INTRED); (iii) both mediation and interaction, or mediated interaction (INTMED); and (iv) only mediation (but not interaction), or pure indirect effect (PIE). This recently proposed approach in Stata, which enables the estimation of the four-way decomposition using parametric or semi- parametric regression models, integrates methods to assign effects to interactions with methods to test mediation. Importantly, *Med4way* command [1] (https://github.com/anddis/med4way) was used to test mediation and interaction of the total effect of poor sleep quality or dementia odds (or status) on the all-cause dementia outcome, with the other exposure becoming an alternative potential mediator/moderator, using Cox PH models for the outcome and OLS for each mediator/moderator. This allowed us to examine those relationships in a bi-directional manner. Four-way decomposition was applied to the total sample, using a common set of exogenous variables, described in the Covariates section. Type I error was set at 0.05 for all analyses.

Additional information is available in supplementary file: appendix-dementia-algorithms-fourwaydecomposition. pdf.

Supplementary literature search and review:

Methods: Utilized PubMed Advanced Search Builder to search for articles within past 5 years, emphasizing 2022 and 2023 articles. Utilized Google to supplement search.

Reviews: Examining sleep quality and mortality; sleep quality and dementia; and dementia andmortality.

- Gao, C., Guo, J., Gong, T. T., Lv, J. L., Li, X. Y., Liu, F. H., Zhang, M., Shan, Y. T., Zhao, Y. H., & Wu, Q. J. (2022). Sleep duration/quality with health outcomes: An umbrella review of meta-analyses of prospective studies. *Frontiers in Medicine*, *8*, 813943. https://doi.org/10.3389/fmed.2021.813943
    - Only the evidence of the association of long sleep with an increased risk of all-cause mortality was graded as highly suggestive.
    - Suggestive evidence supported the associations between long sleep and 5 increased risk of health outcomes (stroke, dyslipidemia, mortality of coronary heart disease, stroke mortality, and the development or death of stroke).
    - Examined 85 meta-analyses.

- Casagrande, M., Forte, G., Favieri, F., & Corbo, I. (2022). Sleep quality and aging: A systematic review on healthy older people, mild cognitive impairment and Alzheimer's disease. *International Journal of Environmental Research and Public Health*, *19*(14), 8457. https://doi.org/10.3390/ijerph19148457
    - The most common aspect compromised in Alzheimer's disease and mild cognitive impairment were REM sleep, sleep efficiency, sleep latency, and sleep duration.
    - These results underline that sleep alterations are associated with cognitive impairment.
    - The frequency and severity of sleep disturbance appear to follow the evolution of cognitive impairment.

- Ono, R., Sakurai, T., Sugimoto, T., Uchida, K., Nakagawa, T., Noguchi, T., Komatsu, A., Arai, H., & Saito, T. (2023). Mortality risks and causes of death by dementia types in a Japanese cohort with dementia: NCGG-STORIES. *Journal of Alzheimer's Disease: JAD*, *92*(2), 487–498. https://doi.org/10.3233/JAD-221290
    - Patients with all types of dementia and mild cognitive impairment had higher mortality rates than those with normal cognition.
    - The most common cause of death was pneumonia, followed by cancer.
    - In the mild cognitive impairment, Alzheimer's disease, and dementia with Lewy bodies groups, older age, male sex, and low cognitive function were common prognostic factors but not presence of apolipoprotein E ε4 allele.

Meta-analyses: Examining sleep quality and mortality; sleep quality and dementia; and dementia and mortality.

- Kwok, C. S., Kontopantelis, E., Kuligowski, G., Gray, M., Muhyaldeen, A., Gale, C. P., Peat, G. M., Cleator, J., Chew-Graham, C., Loke, Y. K., & Mamas, M. A. (2018). Self-reported sleep duration and quality and cardiovascular disease and mortality: A dose-response meta-analysis. *Journal of the American Heart Association*, *7*(15), e008552. https://doi.org/10.1161/JAHA.118.008552
    - Self-reported duration of sleep >8 hours was associated with a moderate increased risk of all-cause mortality.
    - Subjective poor sleep quality was associated with coronary heart disease but no difference in mortality.
    - Divergence from the recommended 7 to 8 hours of sleep is associated with a higher risk of mortality and cardiovascular events.

- Shi, L., Chen, S. J., Ma, M. Y., Bao, Y. P., Han, Y., Wang, Y. M., Shi, J., Vitiello, M. V., & Lu, L. (2018). Sleep disturbances increase the risk of dementia: A systematic review and meta-analysis. *Sleep Medicine Reviews*, *40*, 4–16. https://doi.org/10.1016/j.smrv.2017.06.010
    - Compared with individuals without sleep disturbances, subjects who reported sleep disturbances had a higher risk of incident all-cause dementia, AD, and vascular dementia.
    - The subgroup analysis showed that insomnia increased the risk of AD but not vascular or all-cause dementia.

- Sleep disordered breathing was associated with a higher incidence of all-cause dementia, AD, and vascular dementia.

- Saragih, I. D., Saragih, I. S., Batubara, S. O., & Lin, C. J. (2021). Dementia as a mortality predictor among older adults with COVID-19: A systematic review and meta- analysis of observational study. *Geriatric Nursing (New York, N.Y.)*, *42*(5), 1230–1239. https://doi.org/10.1016/j.gerinurse.2021.03.007
  - The pooled mortality rates of dementia and non-dementia older adults infected with COVID-19 were 39% and 20%.
  - Overall, dementia was the main factor influencing poor health outcomes and high rates of mortality in older adults with COVID-19 infection.
  - Shows that older adults with dementia with COVID-19 infection have a higher risk of mortality compared with older adults without dementia.

Cohort studies: Examining the association between sleep quality and mortality; sleep quality and dementia; and dementia and mortality.

- Garfield, V., Joshi, R., Garcia-Hernandez, J., Tillin, T., & Chaturvedi, N. (2019). The relationship between sleep quality and all-cause, CVD and cancer mortality: The Southall and Brent REvisited study (SABRE). *Sleep Medicine*, *60*, 230–235. https://doi.org/10.1016/j.sleep.2019.03.012
  - Findings suggest that the relationship between sleep quality and mortality may differ by ethnic group.
  - In Europeans, early morning waking was associated with a moderately increased risk of cardiovascular death.
  - In South Asians difficulty falling asleep was related to an increased risk of all-cause mortality.

- Wong, R., & Lovier, M. A. (2023). Sleep disturbances and dementia risk in older adults: Findings from 10 Years of national U.S. prospective data. *American Journal of Preventive Medicine*, *64*(6), 781–787. https://doi.org/10.1016/j.amepre.2023.01.008
  - In the unadjusted model, sleep-initiation insomnia was significantly associated with a 51% increased dementia risk.
  - Adjusted for sociodemographics, sleep-medication usage was significantly associated with a 30% increased dementia risk.
  - Adjusted for sociodemographics and health, sleep-maintenance insomnia was significantly associated with a 40% decreased dementia risk.

- Taudorf, L., Nørgaard, A., Brodaty, H., Laursen, T. M., & Waldemar, G. (2021). Dementia increases mortality beyond effects of comorbid conditions: A national registry-based cohort study. *European Journal of Neurology*, *28*(7), 2174–2184.https://doi.org/10.1111/ene.14875
  - The comorbidity load was associated with increased mortality in both people with and without dementia.
  - Mortality in dementia remained increased, even after adjusting for psychiatric and chronic somatic comorbidities. Our findings suggest that dementia disorders alone contribute to excess mortality, which may be further increased by comorbidities.
  - Findings suggest that dementia disorders alone contribute to excess mortality, which may be further increased by comorbidities.

SUPPLEMENTARY REFERENCE

1. Discacciati A, Bellavia A, Lee JJ, Mazumdar M, Valeri L. Med4way: a Stata command to investigate mediating and interactive mechanisms using the four-way effect decomposition. Int J Epidemiol. 2018. [Epub ahead of print]. https://doi.org/10.1093/ije/dyy236 PMID:30452641
