# Supplementary material for: Poor sleep quality, dementia status and their association with all-cause mortality among older US adults
Source: Aging (Albany NY). 2024 Sep 4;16(17):12138–67. doi: 10.18632/aging.206102 (PMC11424588; doi:10.18632/aging.206102)
Supplement: Supplementary Figure [file aging-16-206102-s002.pdf]

SUPPLEMENTARY FIGURE

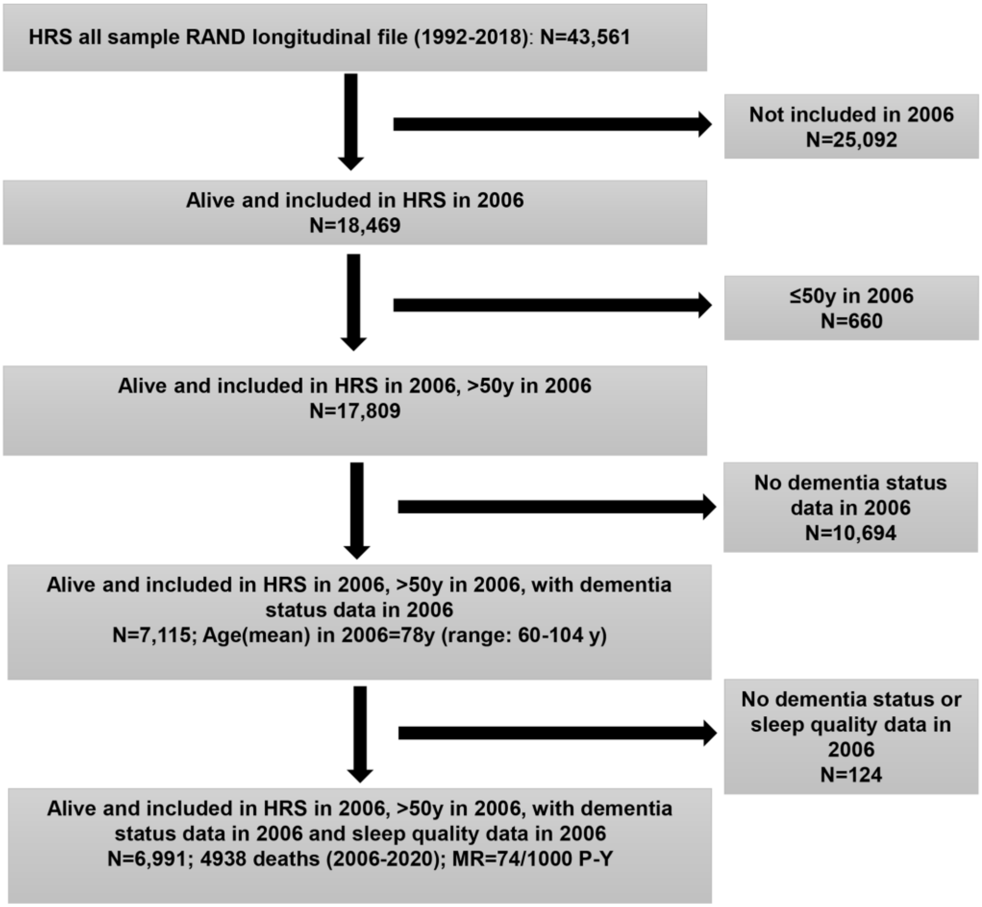

Supplementary Figure 1. Participant flowchart. Abbreviations: HRS: Health and Retirement Study; MR: Mortality Rate; P-Y: Person-Years.
