# Supplementary material for: Poor sleep quality, dementia status and their association with all-cause mortality among older US adults
Source: Aging (Albany NY). 2024 Sep 4;16(17):12138–67. doi: 10.18632/aging.206102 (PMC11424588; doi:10.18632/aging.206102)
Supplement: Supplementary Tables [file aging-16-206102-s003.pdf]

SUPPLEMENTARY TABLES

Supplementary Table 1. Dementia odds (Log$_e$ transformed) and all-cause mortality: four-way decomposition models by poor sleep quality (alternative measure), overall, by sex and by race: HRS 2006–2020[a,b].

Y: All−cause mortality; X: Poor sleep quality score	TE		CDE		INTREF		INTMED		PIE	
	β ± SE	*P*	β ± SE	*P*	β ± SE	*P*	β ± SE	*P*	β ± SE	*P*
**Overall**										
**Reduced Model 1A–1C (*N* = 6,991)**										
X: Hurd	**+0.39460 ± 0.01918**	**<0.001**	**+0.39453 ± 0.01903**	**<0.001**	−0.00014 ± 0.00038	0.72	−3.30e–07 ± 0.00011	0.99	+0.00020 ± 0.00044	0.65
X: Expert	**+0.62019 ± 0.0298**	**<0.001**	**+0.62213 ± 0.02983**	**<0.001**	+0.00044 ± 0.00144	0.76	**−0.00357 ± 0.00156**	**0.022**	+0.00120 ± 0.00081	0.14
X: LASSO	**+0.58189 ± 0.026789**	**<0.001**	**+0.58260 ± 0.02677**	**<0.001**	−0.00025 ± 0.00106	0.81	−0.00099 ± 0.00060	0.27	0.00051	0.29
**Full model 2A–2C (*N* = 6,510)**										
X: Hurd	**+0.50834 ± 0.04784**	**<0.001**	**+0.46549 ± 0.045689**	**<0.001**	**+0.01968 ± 0.00630**	**0.002**	**+0.01734 ± 0.00537**	**0.001**	**+0.00583 ± 0.00204**	**0.004**
X: Expert	**+0.38242 ± 0.03200**	**<0.001**	**+0.35769 ± 0.03106**	**<0.001**	**+0.01078 ± 0.00355**	**0.002**	**+0.00946 ± 0.00281**	**0.001**	**+0.0045349 ± 0.0015896**	**0.004**
X: LASSO	**+0.44499 ± 0.03451**	**<0.001**	**+0.41445 ± 0.03359**	**<0.001**	**+0.01213 ± 0.00403**	**0.003**	**+0.01275 ± 0.00343**	**<0.001**	**+0.005669 ± 0.00184**	**0.002**
**Male**										
**Reduced Model 1A–1C (*N* = 2,931)**										
X: Hurd	**+0.56222 ± 0.04511**	**<0.001**	**+0.55160 ± 0.04485**	**<0.001**	***+0.013821 ± 0.00710***	***0.052***	−0.00520 ± 0.00319	0.10	+0.0020 ± 0.00150	0.19
X: Expert	**+0.60690 ± 0.05047**	**<0.001**	**+0.60239 ± 0.05057**	**<0.001**	+0.00737 ± 0.00622	0.24	−0.0054 ± 0.00406	0.18	+0.00255 ± 0.00224	0.26
X: LASSO	**+0.57335 ± 0.04414**	**<0.001**	**+0.56778 ± 0.04411**	**<0.001**	+0.0074 ± 0.00576	0.20	−0.00389 ± 0.00278	0.16	+0.00202 ± 0.00161	0.21
**Full model 2A–2C (*N* = 2,698)**										
X: Hurd	**+0.41639 ± 0.07245**	**<0.001**	**+0.36523 ±0.068596**	**<0.001**	**+0.040039 ± 0.015321**	**0.009**	+0.0085921 ± 0.0069285	0.22	+0.002532 ± 0.0022419	0.26
X: Expert	**+0.26999 ± 0.047615**	**<0.001**	**+0.24175 ± 0.046239**	**<0.001**	**+0.021059 ± 0.0088441**	0.017	0.0048072 ± 0.0032497	0.14	+0.0023808 ±0.0018317	0.19
X: LASSO	**+0.35083 ± 0.051341**	**<0.001**	**+0.32061 ± 0.050018**	**<0.001**	**+0.021737 ± 0.010039**	**0.030**	+0.005549 ± 0.003598	0.12	+0.0029342 ± 0.0020567	0.15
**Female**										
**Reduced Model 1A–1C (*N* = 4,060)**										
X: Hurd	**+0.37611 ± 0.023086**	**<0.001**	**+0.37427 ± 0.022192**	**<0.001**	+0.002193 ± 0.0026789	0.41	−0.0001658 ± 0.0003734	0.66	−0.0001828 ± 0.0003964	0.65
X: Expert	**+0.63458 ± 0.037328**	**<0.001**	**+0.64323 ± 0.037345**	**<0.001**	**−0.0068712 ± 0.0022929**	**0.003**	**−0.0024374 ± 0.0017279**	0.16	+0.0006561 ± 0.0007173	0.36
X: LASSO	**+0.6017 ± 0.034544**	**<0.001**	**+0.60697 ± 0.034311**	**<0.001**	**−0.0053899 ± 0.0024186**	**0.026**	**+0.0002494 ± 0.000989**	0.80	−0.0001342 ± 0.0005357	0.80
**Full model 2A–2C (*N* = 3,812)**										
X: Hurd	**+0.56999 ± 0.06366**	**<0.001**	**+0.53713 ± 0.061636**	**<0.001**	+0.0016369 ± 0.00547	0.77	**+0.022984 ± 0.0078648**	**0.003**	**+0.0082415 ± 0.00313**	**0.008**
X: Expert	**+0.45958 ± 0.043156**	**<0.001**	**+0.44125 ± 0.042226**	**<0.001**	−0.0015519 ± 0.0034798	0.66	**+0.013771 ± 0.0044423**	**0.002**	**+0.0061107 ± 0.0023974**	**0.011**
X: LASSO	**+0.51303 ± 0.046599**	**<0.001**	**+0.48847 ± 0.045698**	**<0.001**	−0.0020449 ± 0.0037046	0.58	**+0.019134 ± 0.0055133**	**0.001**	**+0.0074635 ±** 0.0028207	**0.008**

**White**										
**Reduced Model 1A–1C (*N* = 5,666)**										
X: Hurd	**+0.40035 ± 0.021778**	**<0.001**	**+0.40084 ± 0.021522**	**<0.001**	−0.0000128 ± 0.0009433	0.99	−0.0000583 ± 0.0002188	0.79	−0.000416 ± 0.0007205	0.56
X: Expert	**+0.65841 ± 0.034181**	**<0.001**	**+0.66013 ± 0.034132**	**<0.001**	−0.0013205 ± 0.0010699	0.22	−0.0013384 ± 0.001134	0.24	+0.0009395 ± 0.0007591	0.22
X: LASSO	**+0.58944 ± 0.029623**	**<0.001**	**+0.59089 ± 0.029593**	**<0.001**	−0.0014574 ± 0.0009838	0.14	+0.0001398 ± 0.0007785	0.86	−0.0001271 ± 0.0007059	0.86
**Full model 2A–2C (*N* = 5,324)**										
X: Hurd	**+0.52799 ± 0.05374**	**<0.001**	**+0.4828 ± 0.051197**	**<0.001**	**+0.019272 ± 0.0071537**	**0.007**	**+0.020357 ± 0.0064565**	**0.002**	**+0.0055594 ± 0.0022126**	**0.012**
X: Expert	**+0.43223 ± 0.03738**	**<0.001**	**+0.4029 ± 0.036271**	**<0.001**	**+0.010697 ± 0.0041516**	**0.010**	**+0.013908 ± 0.0038493**	**<0.001**	**+0.0047164 ± 0.0019462**	**0.015**
X: LASSO	**+0.47268 ± 0.039032**	**<0.001**	**+0.43754 ± 0.037907**	**<0.001**	**+0.012776 ± 0.0047307**	**0.007**	**+0.017013 ± 0.0044407**	**<0.001**	**+0.0053461 ± 0.0021258**	**0.012**
**Black/Hispanic/Others**										
**Reduced Model 1A–1C (*N* = 1,325)**										
X: Hurd	**+0.36329 ± 0.040527**	**<0.001**	**+0.3657 ± 0.040865**	**<0.001**	+0.0004392 ± 0.002962	0.88	−0.0004587 ± 0.0021728	0.83	−0.0023979 ± 0.0027029	0.38
X: Expert	**+0.48137 ± 0.060746**	**<0.001**	**+0.48631 ± 0.060752**	**<0.001**	+0.011991 ± 0.0081179	0.14	***−0.011995 ± 0.006882***	***0.081***	−0.0049335 ± 0.0053335	0.36
X: LASSO	**+0.53417 ±0.06236**	**<0.001**	**+0.53799 ±0.062361**	**<0.001**	+0.0063964 ±0.0064947	0.33	−0.0061 ±0.0053094	0.25	−0.0041111 ±0.0041556	0.32
**Full model 2A−C (*N* = 1,186)**										
X: Hurd	**+0.41074 ± 0.10575**	**<0.001**	**+0.38256 ± 0.10227**	**<0.001**	+0.015644 ± 0.014372	0.28	+0.0061412 ± 0.0073845	0.41	+0.0063943 ± 0.0067089	0.34
X: Expert	**+0.22672 ± 0.061705**	**<0.001**	**+0.21797 ± 0.05989**	**<0.001**	+0.0077981 ± 0.0082535	0.35	+0.0002961 ± 0.0021035	0.89	+0.0006594 ± 0.0046543	0.89
X: LASSO	**+0.34001 ± 0.075128**	**<0.001**	**+0.32957 ± 0.073668**	**<0.001**	+0.0044248 ± 0.0090454	0.63	+0.0014626 ± 0.0026447	0.58	+0.004556 ± 0.00579	0.43

Abbreviations: CDE: Controlled Direct Effect; HRS: Health and Retirement Study; INTMED: Mediated Interaction; INTREF: Interaction Referent; PIE: Pure Indirect Effect; SE: Standard Error. [a]Cox PH regression models with mortality as the main outcome and poor sleep quality as the potential mediator/moderator allowed to interact with the main exposure. Log_e (odds (dementia probability)) using Hurd, expert and LASSO algorithms were the main alternative exposures of interest, sample size *N* = 6,991, four-way decomposition analysis. 1 SD of the "poor sleep quality score" corresponded to 0.72-point higher score. 1 SD of Hurd, expert and LASSO algorithm Log_e (dementia probability) corresponded to 3.93, 3.13 and 2.40, respectively. [b]Exogenous variables are the ones included in Table 2, Models 1A–1D and 2A–2D, as covariates for the reduced and full models, respectively. See Covariates section for detail.

Supplementary Table 2. Poor sleep quality (alternative measure) and all-cause mortality: four-way decomposition models by dementia odds (Log_e transformed), overall, by sex and by race, HRS 2006–2020[a,b].

**Y: All-cause mortality; X: Poor sleep quality score**	**TE**		**CDE**		**INTREF**		**INTMED**		**PIE**	
	**β ± SE**	***P***	**β ± SE**	***P***	**β ± SE**	***P***	**β ± SE**	***P***	**β ± SE**	***P***
**Overall**										
**Reduced model 1A–1C (*N* = 6,991)**										
M: Hurd	**+0.030902 ± 0.015271**	**0.043**	**+0.030249 ± 0.014538**	**0.037**	−0.0009163 ± 0.002958	0.76	+0.00000325 ± 0.000066	0.96	+0.0015668 ± 0.003409	0.65
M: Expert	+0.022314 ± 0.01533	0.14	+0.023319 ± 0.014172	0.10	**−0.014888 ± 0.0040651**	**<0.001**	**−0.001674 ± 0.0007357**	**0.023**	**+0.015558 ± 0.0048172**	**0.001**
M: LASSO	+0.021734 ± 0.015277	0.16	**+0.029001 ± 0.014166**	**0.029**	**−0.012448 ± 0.0040104**	**0.002**	−0.0005014 ± 0.0004548	0.27	+0.0056822 ± 0.0046441	0.22
**Full model 2A–2C (*N* = 6,510)**										
M: Hurd	**−0.071307 ± 0.015996**	**<0.001**	**−0.069697 ± 0.016273**	**<0.001**	**+0.0056935 ± 0.0017507**	**<0.001**	**+0.0039003 ± 0.0011567**	**0.001**	**−0.011204 ± 0.0029377**	**<0.001**
M: Expert	**−0.072455 ± 0.016005**	**<0.001**	**−0.065013 ± 0.016198**	**<0.001**	+0.0008955 ± 0.0012469	0.47	**+0.0038437 ± 0.0010987**	**<0.001**	**−0.012181 ± 0.0030042**	**<0.001**
M: LASSO	**−0.074468 ± 0.015933**	**<0.001**	**−0.065691 ± 0.016257**	**<0.001**	**+0.0023859 ± 0.0013247**	***0.072***	**+0.0045361 ± 0.0011665**	**<0.001**	**−0.015699 ± 0.0032712**	**<0.001**
**Male**										
**Reduced Model 1A–1C (*N* = 2,931)**										
M: Hurd	+0.036394 ± 0.023714	0.13	***+0.03855 ± 0.022696***	***0.085***	**−0.012609 ± 0.0038722**	**0.001**	−0.002007 ± 0.0012411	0.11	**+0.01246 ± 0.0061561**	**0.043**
M: Expert	***+0.041568 ± 0.023999***	***0.083***	+0.026619 ± 0.022592	0.24	***−0.0079929 ± 0.004602***	***0.082***	−0.0020728 ± 0.0015948	0.19	**+0.025015 ± 0.0068767**	**<0.001**
M: LASSO	***+0.040998 ± 0.023893***	***0.086***	+0.031346 ± 0.022755	0.17	**−0.0074983 ± 0.003495**	**0.032**	−0.0017166 ± 0.0012603	0.17	**+0.018867 ± 0.0068946**	**0.006**
**Full Model 2A–2C (*N* = 2,698)**										
M: Hurd	**−0.049922 ± 0.025342**	**0.049**	**−0.054646 ± 0.02556**	**0.033**	**+0.0074653 ± 0.0031169**	**0.017**	+0.0018204 ± 0.0014413	0.21	−0.0045613 ± 0.0034561	0.19
M: Expert	***−0.049416 ± 0.025435***	***0.052***	**−0.051529 ± 0.025562**	**0.044**	**+0.0055075 ± 0.0026007**	**0.034**	+0.0019059 ± 0.0012649	0.13	***−0.0053001 ± 0.0031745***	***0.095***
M: LASSO	**−0.051863 ± 0.025306**	**0.040**	**−0.057227 ± 0.026158**	**0.029**	**+0.01078 ± 0.0043433**	**0.013**	+0.001954 ± 0.0012319	0.11	**−0.0073697 ± 0.0039338**	***0.061***
**Female**										
**Reduced Model 1A–1C (*N* = 4,060)**										
M: Hurd	+0.021244 ± 0.01996	0.29	+0.020074 ± 0.018716	0.28	+0.0036149 ± 0.0047921	0.45	−0.0001155 ± 0.0002588	0.66	−0.0023297 ± 0.0045684	0.61
M: Expert	+0.0092236 ± 0.019975	0.64	+0.01992 ± 0.018151	0.27	**−0.020214 ± 0.006299**	**0.001**	−0.0012756 ± 0.0009076	0.16	+0.010794 ± 0.0067233	0.11
M: LASSO	+0.0088882 ± 0.019948	0.66	+0.025951 ± 0.018025	0.15	**−0.015572 ± 0.0068724**	**0.023**	+0.0001363 ± 0.0005395	0.80	−0.0016275 ± 0.0063951	0.80
**Full Model 2A–2C (*N* = 3,812)**										
M: Hurd	**−0.087193 ± 0.020602**	**<0.001**	**−0.080351 ± 0.021098**	**<0.001**	***+0.0039831 ± 0.0021884***	***0.069***	**+0.0053083 ± 0.0017144**	**0.002**	**−0.016134 ± 0.0044396**	**<0.001**
M: Expert	**−0.089694 ± 0.020571**	**<0.001**	**−0.073317 ± 0.020868**	**<0.001**	**−0.0041846 ± 0.0021176**	**0.048**	**+0.0056334 ± 0.0017336**	**0.001**	**−0.017826 ± 0.0047459**	**<0.001**
M: LASSO	**−0.091803 ± 0.020474**	**<0.001**	**−0.068552 ± 0.020738**	**0.001**	**−0.0076524 ± 0.0023243**	**0.001**	**+0.0068409 ± 0.0018637**	**<0.001**	**−0.022439 ± 0.0049456**	**<0.001**
**White**										
**Reduced Model 1A–1C (*N* = 5,666)**										
M: Hurd	**+0.042615 ± 0.017248**	**0.013**	**+0.045593 ± 0.016644**	**0.006**	−0.0006782 ± 0.00257	0.79	−0.0000424 ± 0.0001336	0.75	−0.0022575 ± 0.0038237	0.56
M: Expert	**+0.035484 ± 0.017316**	**0.040**	**+0.038322 ± 0.016226**	**0.018**	**−0.010111 ± 0.00365**	**0.006**	−0.000566 ± 0.0004904	0.25	+0.0078392 ± 0.0054609	0.15
M: LASSO	***+0.032891 ± 0.017185***	***0.056***	**+0.043853 ± 0.016208**	**0.007**	**−0.010087 ± 0.003663**	**0.006**	+0.0000682 ± 0.0003795	0.86	−0.0009427 ± 0.0052236	0.86

**Full model 2A–2C (*N* = 5,324)**										
M: Hurd	**−0.056391 ± 0.017887**	**0.002**	**−0.061824 ± 0.01847**	**0.001**	**+0.013754 ± 0.0031299**	**<0.001**	**+0.0045156 ± 0.0013674**	**0.001**	**−0.012836 ± 0.0033782**	**<0.001**
M: Expert	**−0.057803 ± 0.017899**	**0.001**	**−0.052722 ± 0.018373**	**0.004**	**+0.0065844 ± 0.0020024**	**0.001**	**+0.0051515 ± 0.001365**	**<0.001**	**−0.016816 ± 0.0036252**	**<0.001**
M: LASSO	**−0.05932 ± 0.017815**	**0.001**	**−0.054199 ± 0.018439**	**0.003**	**+0.0081853 ± 0.0022739**	**<0.001**	**+0.0058666 ± 0.0014602**	**<0.001**	**−0.019172 ± 0.003814**	**<0.001**
**Non-White**										
**Reduced Model 1A–1C (*N* = 1,325)**										
M: Hurd	−0.016372 ± 0.033019	0.62	−0.03019 ± 0.030445	0.32	−0.0018161 ± 0.01184	0.88	−0.0002873 ± 0.001341	0.83	**+0.015922 ± 0.0076197**	**0.037**
M: Expert	−0.02867 ± 0.033247	0.39	−0.02983 ± 0.030918	0.34	***−0.032022 ± 0.016716***	***0.055***	***−0.0070443 ± 0.0039502***	***0.075***	**+0.040226 ± 0.010528**	**<0.001**
M: LASSO	−0.023034 ± 0.033538	0.48	−0.031699 ± 0.029852	0.29	−0.018889 ± 0.015875	0.23	−0.0031746 ± 0.0027188	0.24	**+0.030728 ± 0.010316**	**0.003**
**Full model 2A–2C (*N* = 1,186)**										
M: Hurd	**−0.13108 ± 0.036592**	**<0.001**	**−0.1111 ± 0.037134**	**0.003**	**−0.015845 ± 0.010362**	**0.13**	**+0.0014531 ± 0.0016788**	**0.39**	**−0.0055812 ± 0.0056183**	**0.32**
M: Expert	**−0.13057 ± 0.036569**	**<0.001**	**−0.11974 ± 0.037074**	**0.001**	−0.010308 ± 0.0085493	0.23	+0.0001613 ± 0.0011436	0.89	−0.0006843 ± 0.0048254	0.89
M: LASSO	**−0.13464 ± 0.036457**	**<0.001**	**−0.12457 ± 0.036056**	**0.001**	−0.005761 ± 0.0085282	0.50	+0.0006235 ± 0.0010289	0.54	−0.0049392 ± 0.0061316	0.42

Abbreviations CDE: Controlled Direct Effect; HRS: Health and Retirement Study; INTMED: Mediated Interaction; INTREF: Interaction Referent; PIE: Pure Indirect Effect; SE: Standard Error. [a]Cox PH regression models with mortality as the main outcome and poor sleep quality as the exposure. Log_e (odds (dementia probability)) using Hurd, expert and LASSO algorithms were potential mediators/moderators allowed to interact with the main exposure, four-way decomposition analysis. 1 SD of the "poor sleep quality score" corresponded to 0.72-point higher score. 1 SD of Hurd, expert and LASSO algorithm Log_e (dementia probability) corresponded to 3.93, 3.13 and 2.40, respectively. [b]Exogenous variables are the ones included in Table 2, Models 1A–1D and 2A–2D, as covariates for the reduced and full models, respectively. See Covariates section for detail.
